# The physiologic power of fluid circulation and implications for fluid therapy

**DOI:** 10.3389/fvets.2026.1798503

**Published:** 2026-06-03

**Authors:** William Muir

**Affiliations:** Richard A. Gillespie College of Veterinary Medicine, Lincoln Memorial University, Harrogate, TN, United States

**Keywords:** circulation, hemodynamics, interstitium, lymphatics, volume kinetics

## Abstract

Water is the most prevalent and ubiquitous substance in the body, an essential solvent for all chemical processes and a prerequisite for solute and gas transport. It is reasonable, therefore, that multiple systems and intricate interdependent regulatory mechanisms have evolved to maintain water balance within 1%−2% of body requirements. The overlapping complexity of body fluid regulatory mechanisms provides ample opportunity for healthy animals to tolerate and recover from substantial fluctuations in water volume deficits or excesses, provided that water balance is restored before tissue oxygen and nutrient delivery become irreversibly compromised. Intravenous fluid therapy can restore tissue perfusion in hypovolemic patients, provided the reestablishment of vascular volume and systemic hemodynamic variables are effective in remedying microcirculatory derangements. Inappropriately administered IV fluid, however, results in fluid imbalance, electrolyte and acid–base disorders, fluid accumulation, impaired healing, prolonged recovery, and unfavorable outcomes, especially in patients that are fluid non-responsive or intolerant. Revised and alternative interpretations of circulatory physiology and IV fluid disposition (e.g., volume kinetics) continue to emerge, providing insights on how IV fluids are delivered to avoid adverse effects (e.g., fluid accumulation syndrome) and improve outcomes. Fluid choice, dosage, and the timing of fluid administration are species- and context-dependent and should be individualized based on macro–micro hemodynamic–interstitial–lymphatic concordance, disease-associated changes in fluid disposition, and an understanding of fluid volume kinetics.

## Introduction

**Figure 1:**
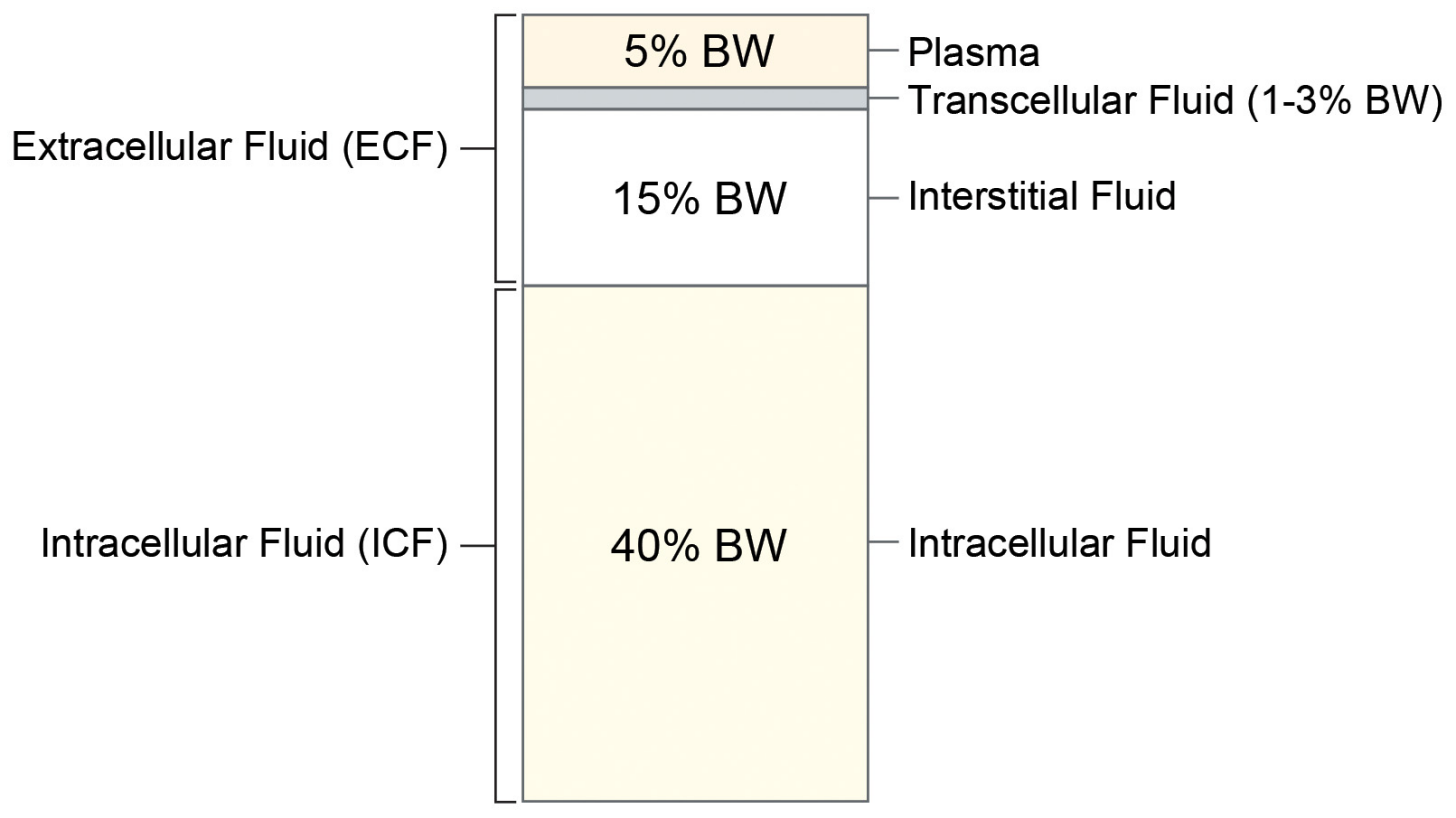
Total body water (TBW) is approximated to be 60%−70% of body weight (BW). TBW is anatomically divided into intracellular (ICF) and extracellular fluid (ECF) compartments, which represent approximately 40 and 25% of body weight (BW), respectively. Most intracellular water (≈65%) resides in skeletal muscle. The ECF is subdivided into interstitial fluid (15% BW), plasma (5% BW), and transcellular fluids (1%−3% BW). Transcellular fluid is contained within epithelial-lined body cavities and includes lymph, cerebrospinal fluid, synovial fluid, ocular fluid, and gastrointestinal fluids, as well as fluid found in pleural, pericardial, and peritoneal cavities. In some ruminants, gastrointestinal tract transcellular water can amount to 15%−35% BW, increasing TBW to greater than 80% of BW.

Life is dependent upon water, and each species has developed genetic, environmental, and behavioral physiologic differences for preserving fluid homeostasis (1, 2). Water accounts for approximately 60 percent or more of body weight in most vertebrates and is the universal diluent for all metabolizing cells (Figure 1) (3, 4). Water imbalance can result in altered temperature regulation, neurological and metabolic dysfunction, electrolyte and acid–base disturbances, organ perfusion deficits, and death. Excessive amounts of imbibed or infused water disturb solute balance (e.g., electrolytes and acid–base balance) and impair tissue and organ perfusion (e.g., fluid accumulation, edema, and effusion) (5). Phylogenetic and environmentally driven species-specific differences have evolved to replace sensible and insensible fluid losses, maintain water balance, and preserve normal body functions (2, 6). Each species has developed qualitatively similar but quantitatively different, and occasionally unique (e.g., neuroendocrine, entero-systemic cycle, spleen, and red blood cells) methods for retaining, redistributing, and restoring (e.g., splenic contraction) vascular volume and tissue perfusion.

The US Food and Drug Administration (FDA) defines a drug as a substance “*intended for use in the diagnosis, cure, mitigation, treatment, or prevention of disease*,” or “*intended to affect the structure or any function of the body*.”^1^ Most IV fluids contain relatively low concentrations of solutes in purified water (e.g., crystalloids ≈99% water; colloids ≥94% water). Pharmacokinetic (PK) studies are required for all new drug applications. The FDA classifies intravenous fluids (i.e., crystalloids, colloids) as drugs (i.e., volume expanders) or components integrated into a particular medical device (e.g., fluid infusion or syringe pump) (7, 8). Notably, hydroxyethyl starch solutions require safety labeling (i.e., boxed warning) indicating that they may increase the risk of kidney injury, bleeding, and mortality^2^, ^3^, ^4^ (9, 10). Blood and blood products (e.g., whole blood, plasma, and blood products: platelets, albumin, and immunoglobulins) are classified as drugs under the category of biologics (see text footnote 2).

More than half of the 15,000 publications investigating the physiology, pharmacokinetics, and prescription of IV fluids cited in PubMed have been published since 2010. Many of these studies have been conducted in rats or humans, are empirical and lack validated objective assessments, and do not address species differences (11). They do, however, provide insights into the types of fluids, experimental methods, and model-based designs that are believed to be important for guiding “*safe and effective dosing recommendations*.”^5^^,^^6^ The translation of fluid maintenance and replacement practices from one species to another does not consider anatomical, physiological, or fluid pharmacokinetic (i.e., volume kinetic) differences and is likely responsible for suboptimal and potentially harmful outcomes, as suggested by recent studies in cats (12, 13). The maintenance and restoration of body fluid balance depend upon a functional understanding of the anatomical and physiological species-specific dynamic processes (e.g., circulatory, interstitial, lymphatic, renal, and neuroendocrine) that maintain fluid balance. Notably, inappropriate IV fluid therapy (e.g., hyper- or hypovolemia) is known to be associated with increased morbidity and mortality, especially in emergency and critical care settings (4, 14, 15).

This review provides a contemporary appraisal of macro–micro hemodynamic–interstitial–lymphatic concordance by discussing the anatomical, physiological, and fluid kinetic elements that participate in fluid circulation and disposition. Relevant models are introduced and revised, and alternative interpretations that extend, expand, and challenge conventional perceptions are presented.

## Models

Models help illustrate, simulate, organize, and describe anatomic, physiologic, and pharmacologic associations that identify, simplify, emphasize, characterize, or predict biological entities, events, relationships, or concepts. Models represent real or hypothetical constructs. Pharmacokinetic (PK) compartmental models are theoretical but help simplify drug behavior, predict drug concentration, and guide drug dosing schedules. Physiologically based pharmacokinetic (PBPK) models employ species-dependent physiologic data (i.e., blood pressures, flow and distribution) and pharmacologic parameters (e.g., drug concentrations, apparent volumes of distribution, and clearances) to represent and simulate real-life effects (see text footnotes 1 and 2) (16, 17). All models are artificial and incorporate assumptions: “*All models are wrong, but some are useful*” (18). Readers are advised to consider model limitations, cautiously appraise the data used to create the model, and avoid models that do not consider species-specific anatomic, physiologic, or functional reality.

## Fluid circulation

**Figure 2:**
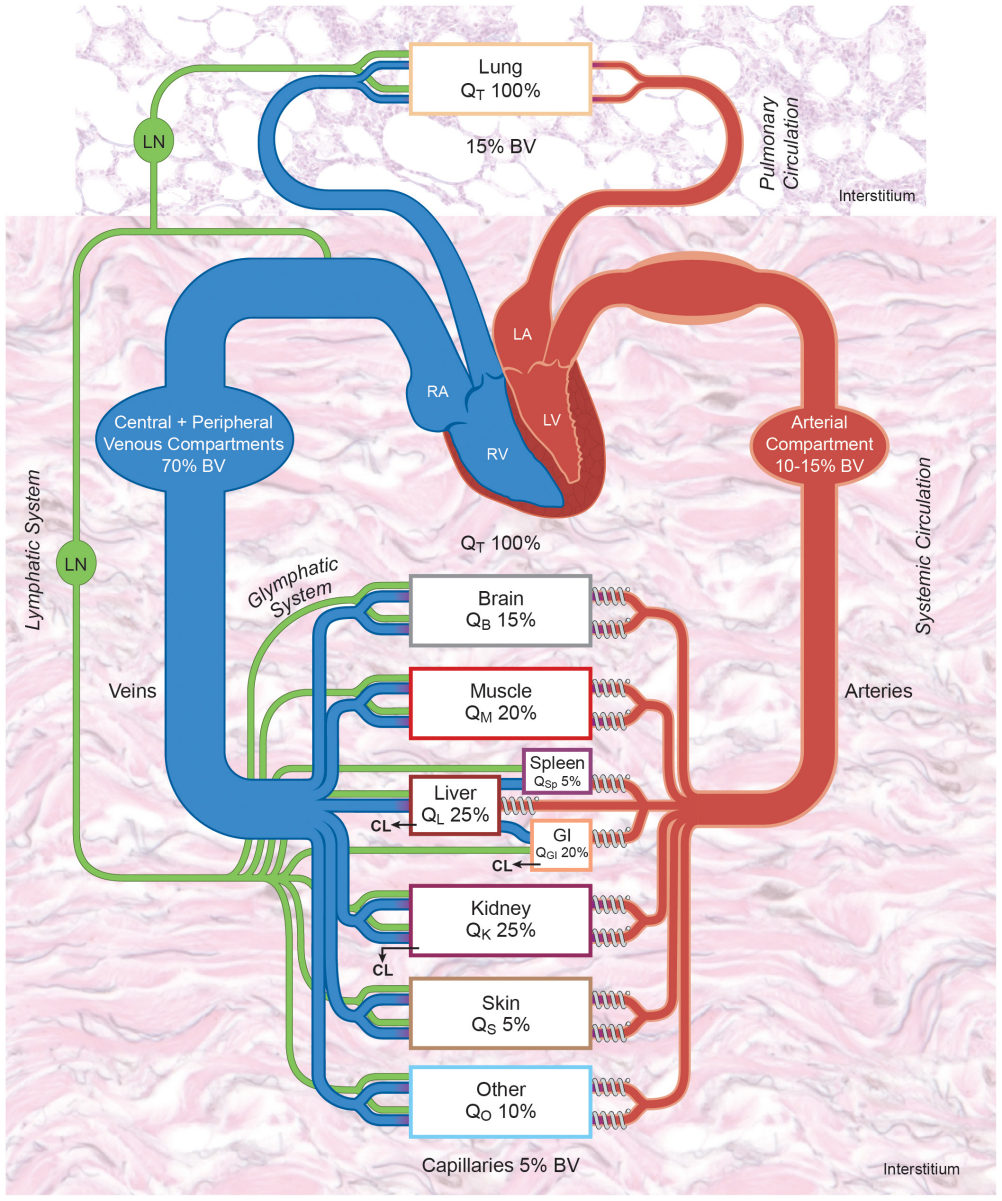
Blood and lymph are circulated throughout the body by closed cardiovascular (red, blue) and open lymphatic and glymphatic (green) systems. Blood is unevenly distributed between the systemic (arteries:10%; capillaries: 5%; veins: 70%; total 85%) and pulmonary (15%) circulations. Systemic organ blood flow (Q_T_ %) is determined by tissue oxygen requirements (e.g., O_2_ demand: DO_2_). Excess water is cleared (CL) as urine, bile, in feces, and sweat (e.g., evaporation from lungs and skin).

The mammalian cardiovascular system (i.e., heart, vessels, blood) circulates blood to two serially connected closed vascular circuits (e.g., pulmonary and systemic). Fluid homeostasis implies the maintenance and regulation of normal fluid volume, the continual movement of fluid throughout the body, and normal electrolyte, osmolarity, and acid–base balance values. Water balance implies matching fluid input to output and is the most important homeostatic function maintaining circulatory volume (19, 20). Contemporary texts emphasize that water balance is dependent upon cardiovascular, respiratory, renal, and neuroendocrine function, as well as physical forces and dynamic adjustments. It is assumed that increases in intravascular volume (i.e., blood volume) improve cardiovascular function (i.e., ability to circulate blood), cardiac performance (i.e., cardiac output), blood flow distribution, and oxygen delivery (DO_2_) to tissues (20, 21). This can only occur or become beneficial, however, in patients that are fluid responsive (22), fluid tolerant (23), and have maintained hemodynamic synergy (i.e., coherence) between macro and micro (e.g., vessels < 100 μm in diameter), interstitial, and lymphatic circulatory components (Figure 2) (19, 24, 25).

### Cardiovascular system

The macrocirculation includes all the major conduit vessels responsible for providing the pressures and flows that transport blood to and from the microcirculation. High-pressure, low-volume, low-compliance arteries contain approximately 10% of the total blood volume, while low-pressure, large-volume, highly compliant veins contain approximately 70% of the total blood volume (26–28). The heart and large blood vessels (i.e., macrocirculation) transport blood to (e.g., large arteries) and from (e.g., large veins) organ-specific capillary beds (i.e., microcirculation) where nutrients (e.g., O_2_) are exchanged for waste products (e.g., CO_2_).

Introductory descriptions of hemodynamics focus on macrohemodynamics, where the heart serves as a pressure (P) generator (e.g., cardiocentrism) and is a primary determinant of blood flow (Q) (29–31). Macrocirculatory hemodynamic events are traditionally described in terms of the hydraulic equivalent of Ohm's law for electrical circuits (i.e., E = I × R): where ΔP is substituted for voltage: E, Q is substituted for current flow: I, and R is determined by their interaction (i.e., ΔP/Q) (30). Heart contraction (e.g., pumping) is considered responsible for producing P (e.g., pressure propulsion) that generates Q, similar to the effects of voltage on current flow (Equations 1 and 2). Total body Q is cardiac output (CO; Equation 3) (30). Darcy's law (e.g., Equation 4) incorporates fluid viscosity, intrinsic permeability, gravitational effects, and vascular geometry, making it more suitable for describing Q in porous microcirculatory elements, where K is hydraulic conductivity incorporating internal radius (r) and dynamic viscosity (η), A is cross-sectional area, and l is length (32, 33). Hydraulic resistance (R) is inversely proportional to K in Darcy's law. Substituting R for K in Darcy's equation produces the Hagen–Poiseuille equation (e.g., where R = 8ηL/πr^4^), a more familiar and nuanced estimate of fixed laminar Q in the macro (i.e., comparatively non-porous) circulation (Equation 5).

Hydraulic equivalent of Ohm's Law


$$\begin{array}{l} \text{ΔP}=\text{Q x R} \end{array}$$ (1)

Rearrangement of Equation 1


$$\begin{array}{l} \text{Q}=\text{ ΔP}/\text{R}\\ \text{Q}=\text{CO} \end{array}$$ (2)

$$\begin{array}{l} \text{Q}=\text{ ΔP}/\text{R} \end{array}$$ (3)

Darcy's Law


$$\begin{array}{l} \text{Q}=-\text{KA x ΔP}/\text{Δl} \end{array}$$ (4)

Hagen–Poiseuille Equation


$$\begin{array}{l} \text{Q}=\text{ΔP }\pi \;{\text{r}}^{\text{4}}/\text{8}\eta \text{l} \end{array}$$ (5)

The cardiocentric view of Q was challenged in the mid-1950s when experiments conducted in anesthetized dogs on cardiopulmonary bypass suggested that pressure differences produced by “the quantity of available blood” in a “venular storage pool” (e.g., indicated by mean circulatory filling pressure: Pmcf), “back pressure from the right atrium” (e.g., Pra), and venous resistance (R_VR_) determine venous return (e.g., VR) and CO (e.g., VR = CO), since “the heart can only pump what it receives” (34). The Pmcf or its surrogate, mean systemic filling pressure (e.g., Pmsf), represents the average pressure throughout the entire circulatory system when the heart is stopped (35, 36), ignores the heart and pulmonary circulation, and posits that right atrial pressure (e.g., Pra) is a dependent determinant (i.e., back pressure) opposing VR (Equation 6) and, therefore, CO (34, 35).


$$\begin{array}{l} \text{CO}=\text{VR}=\text{Pmsf}-\text{Pra}/{\text{R}}_{\text{VR}} \end{array}$$ (6)

This frequently cited, and often misinterpreted, theory of what determines CO is based on Ohmic/Poiseuillian justifications. Pra is not the only cause (i.e., independent variable) of VR in a closed circulation but rather its effect (37, 38). Venous return is dependent upon the coordinated interaction of the entire cardiovascular system (37). It is the heart, coupled with blood volume and its distribution, that is responsible for Pmsf (38). Interpretations relying on measurements of Pmsf to explain changes in CO have confused the physiologic relationship between Pra and VR in the intact circulatory system (37, 38). As one author opined, Pmsf–Pra/R_VR_ “was never about Pmsf physically driving venous return; it was about how intravascular volume distributes among compliant compartments in accordance with their flow-dependent distending pressures, arbitrarily expressed relative to Pra rather than arterial pressure” (38). Although venous return curves provide a mathematical exercise on how VR and CO are interrelated (39), they confuse and divert attention from the importance of the heart as an energy source (40, 41).

Acutely adding fluid to the venous circulation increases Pa in proportion to preload-dependent increases in cardiac contractile function, baroreceptor reflexes, and changes in venous compliance. Mean systemic filling pressure should be considered a “weighted mean of elastic recoil pressures in all systemic vascular beds,” not a pressure somewhere upstream from Pra (38, 40). An increase in Pmsf could represent a change in blood volume, cardiac function, redistribution of blood from arterial to venous vessels, a decrease in venous capacitance, or a decrease in venous compliance (i.e., increased elastance). Without measures of total blood volume and changes in vascular tone, the significance of changes in Pmsf is difficult to interpret.

**Figure 3:**
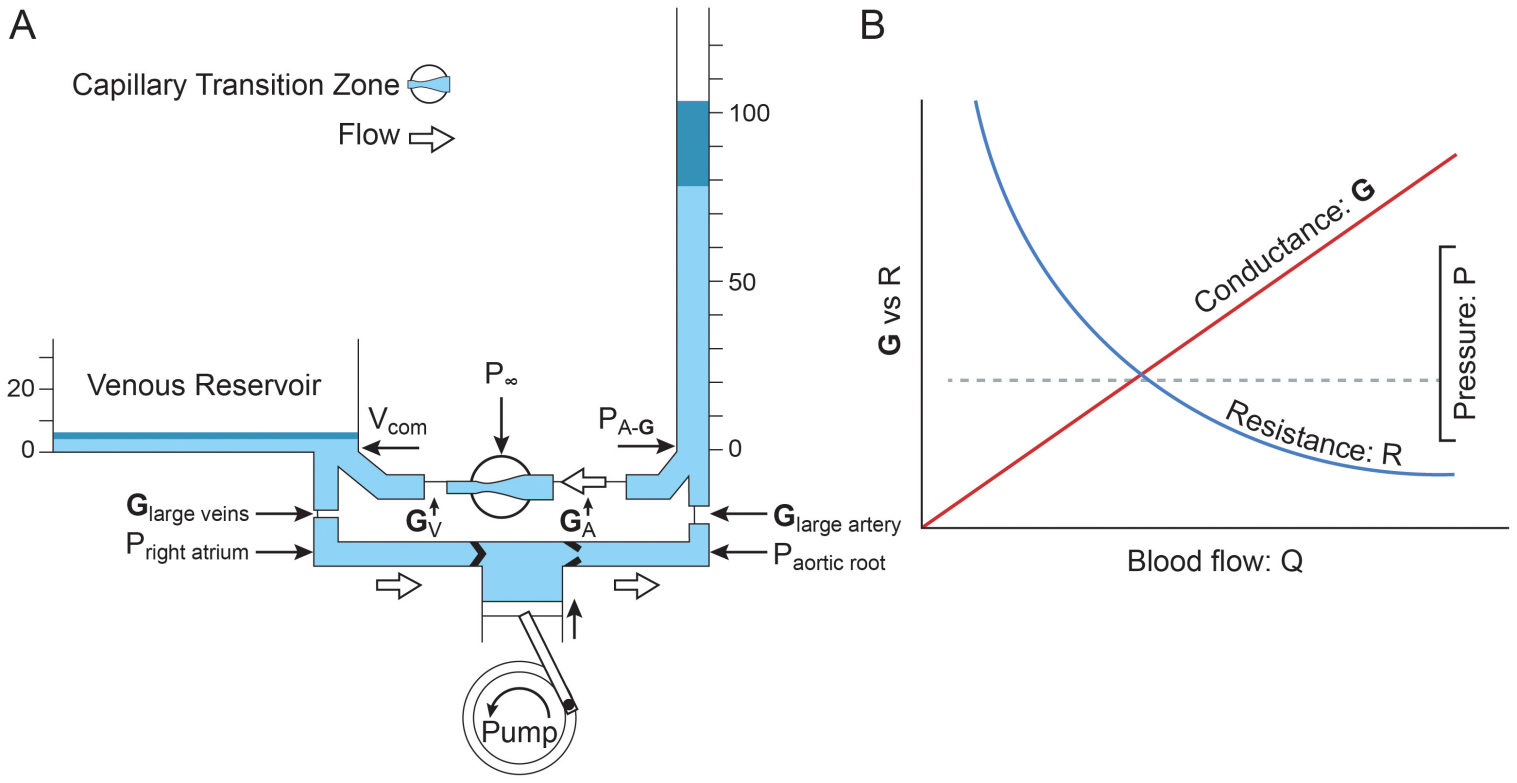
**(A)** Hydraulic model of the systemic circulation with emphasis on changes in vascular compliance (V_com_) and conductance (G). Blood moves from high-pressure (P_A_), low-compliance arteries through a pressurized (P∞) capillary transition zone to a low-pressure, high-compliance venous reservoir, and is returned via large veins to the right atrium. Note that increases in blood volume or decreases in V_com_ increase venous excess and cardiac output (modified from reference (42)). **(B)** Relationship of vascular conductance (red line: G) and resistance (blue line: R) to blood flow (Q). Note: At constant pressure (P), G is directly and linearly related to Q (G = Q/P). If pressure changes, the relationship becomes non-linear because both Q and P can change at different rates.

Other investigators have proposed that conductance, capacitance, and compliance are underemphasized but more accurate descriptors of non-Newtonian (i.e., blood) flow in dynamic fluid compartments (42, 43). These parameters and resultant models focus attention on fluid distribution, compartmental volumes (e.g., capacitance), distensibility (e.g., compliance), and ease of blood flow (e.g., conductance: G; Figure 3A) (42). The terms capacitance and compliance (C), although often used interchangeably, have different meanings. Capacitance (C_cap_) describes the volume (V) contained in a vessel or group of vessels at a fixed P (i.e., V at a fixed P), while compliance (C_com_) describes a change in V (i.e., ΔV) in response to a change in P (e.g., ΔP; Equation 7).


$$\begin{array}{l} \text{C}=\text{ ΔV}/\text{ΔP} \end{array}$$ (7)

Compliance, the reciprocal of elastance (E: E = ΔP/ΔV or 1/C), describes the ability of a fluid space or compartment to change its volume as pressure within the space changes. Increased compliance implies that the space or compartment is easily distended (e.g., volume increases) by a relatively small increase in pressure (e.g., veins: high capacitance; low elastance). Compliance depends on the initial volume of the vessels or tissues and their smooth muscle composition. Conductance (G) is the reciprocal of R (e.g., Equation 8) and is a proportional linear indicator of Q (G = Q/P: Equation 9) or CO (Equation 10). Conductance is linear to flow, but resistance is non-linear due to changes in vessel diameter or turbulent blood flow (Figure 3B). This relationship suggests that G is a more clinically reliable predictor of changes in flow or CO than R, particularly at low and high blood flows (43).


$$\begin{array}{l} \text{G}=\text{1}/\text{R}=\;\pi \;{\text{r}}^{\text{4}}/\text{8}\eta \text{l} \end{array}$$ (8)

$$\begin{array}{l} \text{G}=\text{Q}/\text{ΔP}\\ \text{Q}=\text{CO} \end{array}$$ (9)

$$\begin{array}{l} \text{Q}=\text{ΔP x G} \end{array}$$ (10)

The determination of systemic vascular conductance has been shown to provide a “fundamentally more meaningful” (42) assessment of sympathetic modulation of LV contractility, venous capacitance, and CO than changes in R (42–45).

Recent theories and models of circulation posit that (1) the heart is not merely a propulsion pump (e.g., muscle contraction) but an impedance or hydraulic ram pump (e.g., using kinetic energy to move blood), (2) CO is primarily determined by tissue metabolic demand (i.e., DO_2_), and (3) arterial pressure is determined by BV and vascular (e.g., venous and arterial) Ccap and Ccom not systemic vascular resistance (e.g., R) (46–49). This shift from a cardiocentric (e.g., centrifugal) view to a tissue-centric (i.e., centripetal) perspective on circulation focuses attention on blood volume, capacitance, compliance, and therapies that enhance blood flow (e.g., Aortix^®^, https://www.procyrion.com) and tissue oxygen requirements. Future IV fluid studies should determine their effects on the effective vascular volume (i.e., the amount of blood actively perfusing tissues), venous Ccap and Ccom, and non-cardiac therapies that optimize blood flow and tissue oxygen delivery (50, 51).

What causes blood to flow and fluid to circulate continues to be a topic of investigation, debate, and confusion (29, 45, 49, 52–58). Both P and Q are governed by fundamental physical laws, but determining which factor is responsible (i.e., independent variable) for producing a change in subsequent events (i.e., dependent variable) presents conceptual challenges because of the choice of a starting point within the circulation (e.g., arterial or venous), the dynamic complexity of multiple interacting components, and whether the heart (e.g., cardiocentric view) is the only source of Q (Box 1) (29, 45–58).

Box 1 Factors that generate or facilitate blood flow and fluid circulation.

1. Heart
  a. Pressure propulsion and/or suction pump
  b. Impedance (e.g., hydraulic ram) pump
2. Venous excess
3. Thoracic pump
4. Muscle contraction
5. One-way valves
6. Metabolic demand
7. Blood momentum
8. Gravity (e.g., siphon principle)
9. Negative interstitial pressure
10. Interstitial dynamic transport (e.g., dynamotaxis)
11. Lymphatic pumping
12. Autonomous flow: Electrostatic repulsion (e.g., negatively charged RBCs and GCX)

The Ohmic/Poiseuillian description of the cardiovascular system should be considered an introductory and oversimplified interpretation of macrocirculatory events (e.g., P, Q, and R) due to invalid assumptions (e.g., non-pulsatile flow, constant viscosity, rigid tubes, and focus on resistance), the use of static (i.e., non-dynamic) hemodynamic descriptors, and incomplete knowledge of biofeedback mechanisms (56, 57, 59). Fluid infusion-induced macrocirculatory changes do not always predict or ensure beneficial microcirculatory and cellular responses but must be employed with therapies (e.g., astragaloside) and monitoring techniques that do (60–63).

Forthcoming physiological studies should adopt a holistic dynamic evaluation of macro–micro hemodynamic interactions (e.g., Doppler Peripheral Venous Duplex Assessment), fluid responsiveness (e.g., pulse pressure variation: PPV, stroke volume variation: SVV; pulse variability index: PVI; microvascular flow imaging: MVFI), and fluid tolerance (e.g., point of care ultrasound: POCUS), particularly in critically ill patients (64–68).

### Microcirculation, interstitium, and lymphatic system

The microcirculation (e.g., vessels < 100 μm) includes terminal arterioles, metarterioles, capillaries, post-capillary venules, and the intra- and peri-vascular constituents (e.g., endothelial surface layer (ESL) and pericytes) that modulate tissue perfusion, capillary filtration, and tissue perfusion. Collectively, transcapillary fluid exchange, interstitial physical properties (e.g., C_cap_ and C_com_), and lymphatic pumping maintain and regulate extracellular fluid volume, fluid balance, and fluid circulation (19, 32).

### Transcapillary fluid exchange

Identification and interpretation of the factors that influence fluid and solute transport across capillary membranes have evolved since Starling proposed a balance between hydrostatic and osmotic forces (69). The identification of the glycocalyx and sub-glycocalyx space, elucidation of mechanisms responsible for volume-dependent changes in interstitial physical properties (e.g., C_com_), and the recognition that the lymphatic system is the principal method for the return of filtered fluid to the venous circulation have revised the interpretation of fluid filtration (70–72).

**Figure 4:**
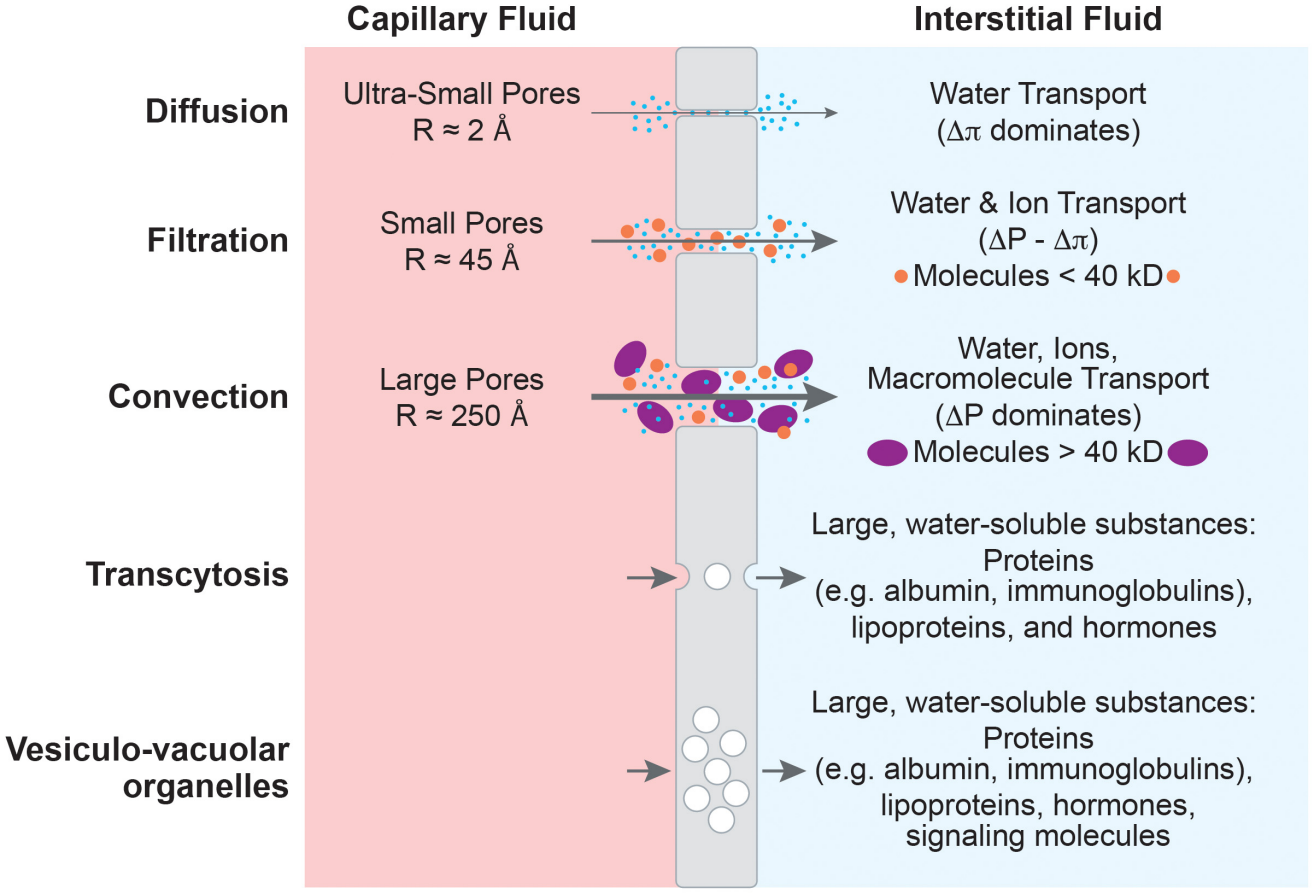
Transcapillary fluid and solute transport mechanisms include diffusion, filtration, convection, transcytosis, and vesiculo-vacuolar organelles (VVAs).

Capillaries (e.g., continuous, fenestrated, and sinusoidal) and postcapillary venules are the principal sites for gas, fluid, and solute exchange. Capillaries are single-cell vascular membranes that utilize diffusion (i.e., concentration gradients), filtration (e.g., bulk flow), transcytosis [e.g., transendothelial transport: vesiculovacuolar organelles (VVOs)], and active transport (i.e., ATP-dependent: Na^+^, K^+^) as exchange mechanisms between intravascular (i.e., plasma) and interstitial fluid compartments (Figure 4) (73–75). Plasma solutes, mainly sodium, chloride, bicarbonate ions, and plasma proteins (e.g., albumin), contribute to plasma osmolarity. Albumin, a semipermeable large molecule (>40 kDa), constitutes less than 0.5% of plasma osmolarity (e.g., 280–315 mOsm/L) but contributes approximately 80% of the colloid osmotic (i.e., oncotic) pressure (e.g., π: 18–24 mm Hg), one-third (e.g., 6–8 mm Hg) of which is due to the Gibbs–Donnan effect (i.e., large, impermeant charged molecules attract Na^+^ and Cl^−^, creating an osmotic pressure difference) (76, 77). Transcapillary solute flux (Equation 11) is determined by


$$\begin{array}{l} \text{Js}=\;\omega \text{Δ }\pi \;+\text{J}\nu \;\left(\text{1}-\;\sigma \;\right)\text{c} \end{array}$$ (11)

where Js is solute flux, ω is the solute permeability coefficient, Δπ is the oncotic pressure difference across the membrane, c is the average concentration ([c1 + c2]/2) of solute across the capillary membrane, σ is the Staverman reflection coefficient (0–1), and J*v* is the net volume flux produced by a balance of capillary hydrostatic (P_c_; pushing force) and oncotic (π; pulling force) pressures (Starling forces), ω*ΔΠ* represents diffusion, and Jv (1–σ)c represents solvent drag (78).

The luminal side of all capillaries is lined by an endothelial surface layer (ESL). The ESL contains water-soluble molecules (e.g., proteins, adhesion molecules, and antithrombin), platelets, leukocytes, regulatory enzymes (e.g., antioxidants), nanobubbles (e.g., O_2_, CO_2_, and N_2_), and a membrane-bound gel (i.e., glycocalyx: GCX) (79–81). The GCX varies in composition among organs, occupies a variable percentage of the intravascular volume based on capillary type (e.g., continuous ≈15%; fenestrated ≈15%; sinusoidal ≈4%), and is composed of endothelial membrane-bound negatively charged glycoproteins, proteoglycans, and glycosaminoglycans (GAGs; e.g., hyaluronic acid, heparin sulfate, and chondroitin sulfate) (82–84). The GCX acts as a porous protective covering (e.g., filter) that impedes diffusion by steric hindrance and electrostatic repulsion, restricts diffusion of plasma proteins (e.g., albumin), modulates capillary membrane permeability [e.g., hydraulic conductivity (K)], senses shear stress (e.g., triggers NO production) (82), and protects endothelial cells from circulating immune cells, cytokines, and oxidants (85–90). Bulk fluid flow (i.e., convection) and the transport of larger solutes across capillary membranes take place through large diameter (e.g., >5–7 nm) pores (e.g., fenestrations and endothelial intercellular clefts) (91–93). Fenestrated capillaries (e.g., kidneys, intestinal tract, pancreas, and endocrine glands) have larger pores (e.g., >7 nm), while sinusoidal capillaries (e.g., liver, spleen, bone marrow, and lymph nodes) have gaps (e.g., >60 nm) between endothelial cells (74, 93–95). Limited studies have simultaneously investigated the effects of solute concentration, polarity, and electrical charge on solute diffusion vs. convection (e.g., Peclet number) in various clinical contexts (e.g., trauma and sepsis) (96–98). The qualitative and quantitative contributions of the GCX on transcapillary fluid and solute transport in different clinical scenarios (e.g., anesthesia, hypoxia, trauma, and sepsis) remain unresolved and requires continued study (96–102).

Continuous capillaries (e.g., heart, lung, muscle, brain, skin, and fat) are the most abundant (85%−90%), smallest (e.g., 5–10 μm diameter), and least likely type of capillary to reabsorb filtered (e.g., interstitial) fluid. They are characterized by a prominent GCX (g), continuous basement membrane, and a comparatively limited number of small pores (e.g., < 5 nm). Diffusion and filtration are their primary methods for capillary exchange. Continuous capillaries do not reabsorb fluid (e.g., no reabsorption rule) during steady-state conditions (71, 103). Large (e.g., M.W. >30–40 kDa) water-soluble solutes and charged (i.e., polar) molecules have difficulty diffusing across the endothelium, and those that are greater than 70 kDa or have a hydrodynamic radius greater than 4–5 nm are impermeable and depend on transcytosis for entry into the interstitium (73, 74, 93).

Transvascular fluid flux (Jv) was initially described in terms of differences in hydrostatic and oncotic pressures as represented by the Starling equation (Equations 12 and 13; (69)):


$$\begin{array}{l} \text{Jv}=\text{K}\left[\left({\text{P}}_{\text{c}}-\;{\text{P}}_{\text{i}}\right)\;-\;\sigma \;\left({\;\pi \;}_{\text{c}}-{\;\pi \;}_{\text{i}}\right)\right] \end{array}$$ (12)

$$\begin{array}{l} \text{Jv}=\text{K}\left(\text{ΔP}-\;\sigma \text{ Δ }\pi \;\right) \end{array}$$ (13)

where capillary (c) and interstitial (i) hydrostatic (P) and oncotic (π) pressure differences (Δ), membrane filtration coefficient (K = surface area: S × hydraulic conductance: Lp), and the membrane's reflection coefficient (σ) for larger solutes (e.g., proteins; σ: 1= impermeable; 0 = freely permeable) (104–106). Oncotic (i.e., colloid osmotic) pressure is produced by large molecules (e.g., albumin) and counteracts hydrostatic pressure by pulling water from areas of low to high solute concentration. Capillary hydrostatic pressure (Pc) is the primary determinant of capillary recruitment, distention (i.e., surface area), and Jv during normal conditions (70, 71). Increases in pre-capillary (a) or post-capillary (v) pressure (e.g., Pa and Pv) cause an increase in Pc that is modified by the pre- to post-capillary resistance (e.g., Ra/Rv) or, alternatively, conductance (e.g., Gv/Ga) ratio (43, 44, 107). A decrease in Ga caused by arteriolar constriction decreases Pc and Jv and vice versa. A decrease in Gv increases Pc and interstitial fluid accumulation. Notably, venules have a greater number of more diffusely distributed and more sensitive alpha-1 and alpha-2 receptors than arterioles and play a greater role in determining Pc and Jv.

**Figure 5:**
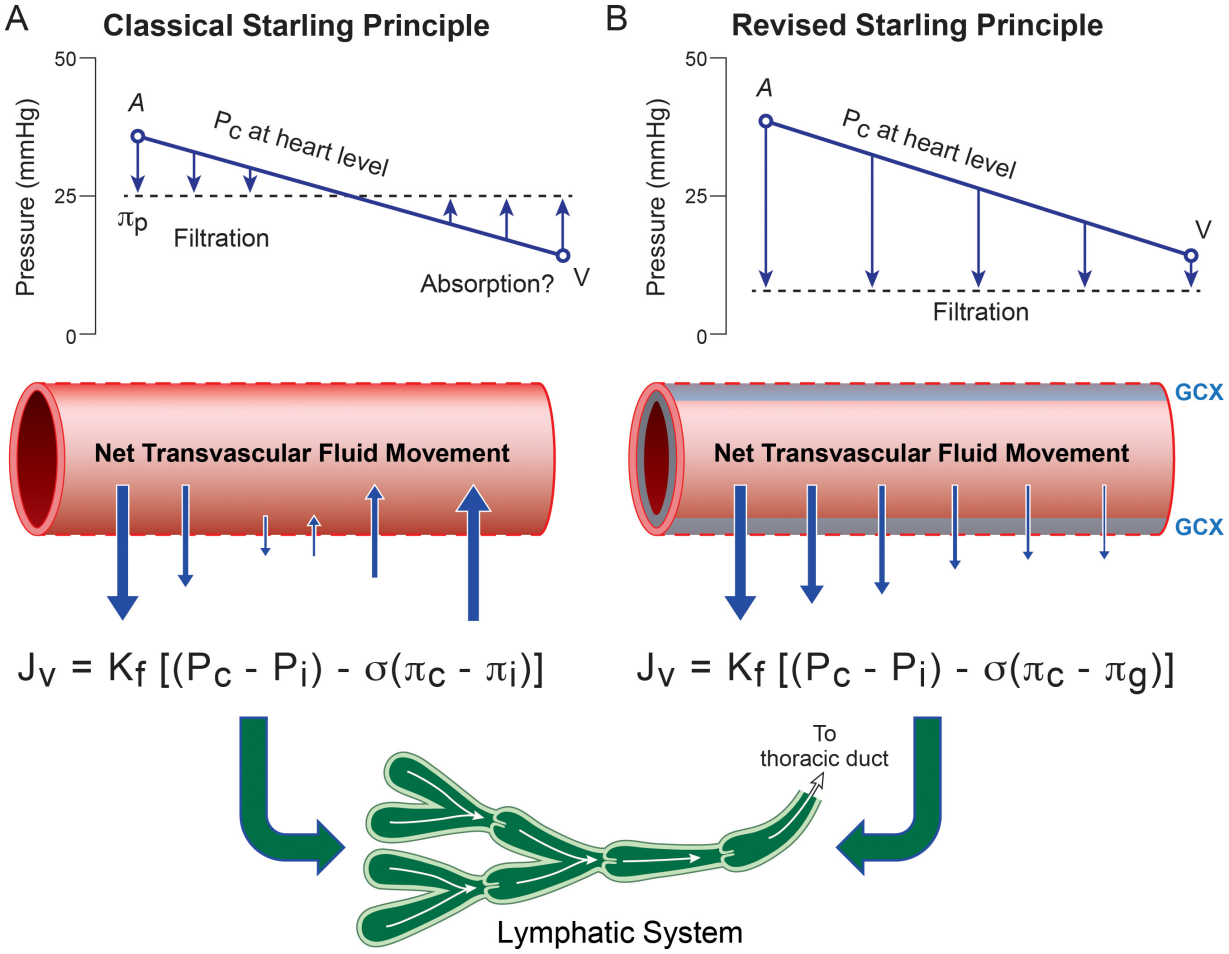
Classical and revised Starling principles for transcapillary fluid flux (Jv). Intracapillary (c) and interstitial (i) hydrostatic (P) and osmotic (π) forces (i.e., Starling forces) determine Jv and are modified by the filtration coefficient [K_f_ = surface area × hydraulic conductance (Lp)] and σ (solute reflection coefficient). **(A)** The Classical Starling Principle states that Starling forces filter fluid at the arteriolar end of the capillary and reabsorb fluid at the venular end of the capillary. **(B)** The Revised Starling Principle posits that fluid is filtered throughout the length of the capillary and not reabsorbed at the venular end of the capillary during steady-state conditions. The lymphatic system is responsible for returning filtered fluid (i.e., lymph) to the systemic circulation. Capillary hydrostatic pressure (Pc) is the primary determinant of J_v_ in both the Classical and Revised Starling Principles.

Starling theorized that capillary hydrostatic pressure (P_c_) predominated at the arterial end of the capillary, promoting fluid filtration (Jv), and that capillary oncotic pressure (π_c_) predominated at the venous end of the capillary, promoting fluid reabsorption (Figure 5A) (69). Subsequent research, however, determined that (1) continuous capillaries normally filter fluid into the interstitium throughout their length; (2) the sub-GCX space (π_g_) is relatively protein-free and opposes fluid filtration in continuous capillaries; (3) Jv is much lower than originally theorized; and (4) the lymphatic system is responsible for returning the majority of the filtered fluid (i.e., lymph) to the venous circulation (108, 109). These discoveries extended Starling's principle (RSP) and revised the Starling equation (Figure 5B; Equation 14) (107, 109):

Revised Starling equation (RSP)


$$\begin{array}{l} \text{Jv}=\text{K}\left[\left({\text{P}}_{\text{c}}-{\text{P}}_{\text{i}}\right)-\;\sigma \;\left({\;\pi \;}_{\text{c}}-{\;\pi \;}_{\text{g}}\right)\right] \end{array}$$ (14)

**Figure 6:**
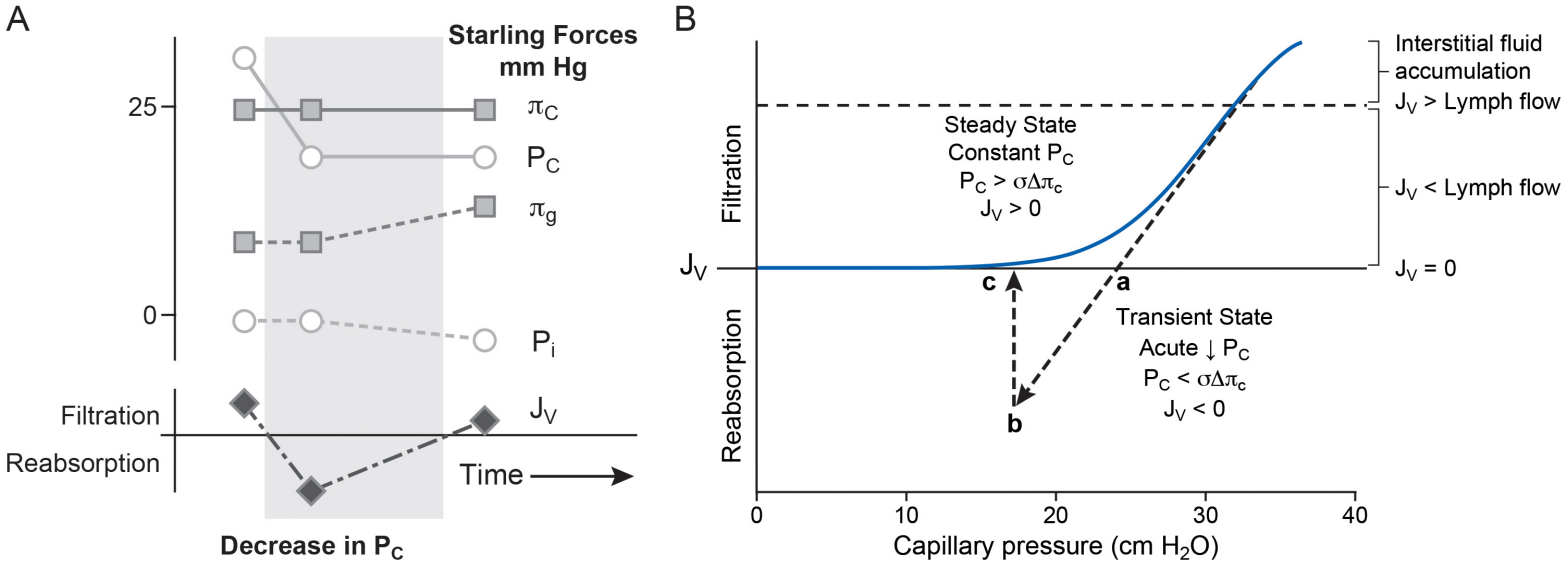
**(A)** Time-dependent changes in J_V_ and Starling forces caused by an acute decrease in Pc. The duration of the resorptive phase (shaded area) varies among tissues (modified from reference (112)). **(B)** Acute decreases in Pc result in J_v_ < 0 and fluid reabsorption (a–b: downward dashed black arrow). Capillary and interstitial readjustments in Starling forces return J_v_ < 0 (b–c: upward dashed black arrow) at steady state. Interstitial fluid accumulates when J_v_ exceeds lymph flow (dashed horizontal black line).

The RSP theorizes that the low sub-GCX oncotic pressure (π_g_) opposes filtration between the plasma and the interstitium (π_g_ < π_i_ < π_c_). The GCX regulates and limits Jv. Transvascular fluid flux is primarily maintained by capillary hydrostatic pressure (Pc). The GCX is responsible for maintaining a relatively protein-free sub-glycocalyx space (e.g., ultrafiltrate) that preserves a colloid osmotic pressure gradient, which limits intravascular (i.e., capillary) fluid loss, primarily through intercellular clefts. Albumin, the body's major water-soluble colloid, provides most of the plasma (70%−80%) and interstitial (50%) oncotic pressure (π_i_) and enters the interstitium by bulk flow (i.e., convection) through endothelial paracellular clefts (e.g., intercellular junctions or gaps) and transcytosis (110, 111). Capillaries experiencing low blood pressure or an acute increase in π_c_ transiently reverse Jv, but fluid is not reabsorbed (e.g., no reabsorption rule) during steady-state conditions (Figures 6A and B) (112). Capillary filtration stops when the Pc falls below π_c_ (e.g., ≈20–25 mm Hg) or when π_c_ or P_i_ increases above P_c_. Studies in anesthetized humans have shown that hemodilution occurs when Pa is acutely decreased by 20% or more below baseline (113), implying that changes in Starling forces initiate fluid reabsorption until steady-state conditions are restored (112). If the GCX is significantly damaged or removed, the primary factor preventing fluid from being reabsorbed is the loss of the transcapillary oncotic pressure difference (π_c_-π_i_). Protein leakage into the sub-GCX space and interstitium results in increased interstitial oncotic pressure (106). Fluid reabsorption of interstitial fluid is reduced or eliminated because the oncotic pressure of the plasma becomes less important in restraining fluid loss (i.e., transcapillary oncotic pressure is reduced), leading to tissue fluid accumulation that now depends upon lymphatic function (108, 109).

A principal goal of the circulatory system and fluid circulation is the optimization of tissue DO_2_. Approximately 5% of the total blood volume resides in capillaries, but only 20%−30% of the body's capillaries are perfused at any given time (114). This implies that dramatic and potentially unsustainable increases in heart rate and cardiac output would be required to maintain tissue perfusion if all capillaries were perfused simultaneously (115, 116). Resting tissue oxygen partial pressure (PtO_2_) varies considerably among different organs, is maintained within a normal functional range (i.e., physioxia), and is regulated by each tissue's metabolic rate (i.e., oxygen requirements) (117–119). Systemic decreases in PtO_2_ (e.g., anemia or hypoxia) trigger vasodilation, while increased PtO_2_ causes vasoconstriction (116). Tissue PtO_2_ values outside each organ's normal range result in anaerobic metabolism (e.g., low PtO_2_) or oxidative stress (e.g., high PtO_2_), leading to the production of tissue-damaging oxygen free radicals, mitochondrial dysfunction, and abnormal cell function (120). Homeostatic autoregulatory mechanisms and vascular tone modulation normally serve to optimize tissue PtO_2_ and reduce oxidative stress. The mean intercapillary distance in most tissues is approximately 50 μm or lower in metabolically active tissues (e.g., heart and kidney ≈15 μm). Most of the oxygen (e.g., 60%−70%) is released by parenchymal arterioles (70%) rather than capillaries and can diffuse radially 100–200 μm (121–123).

**Figure 7:**
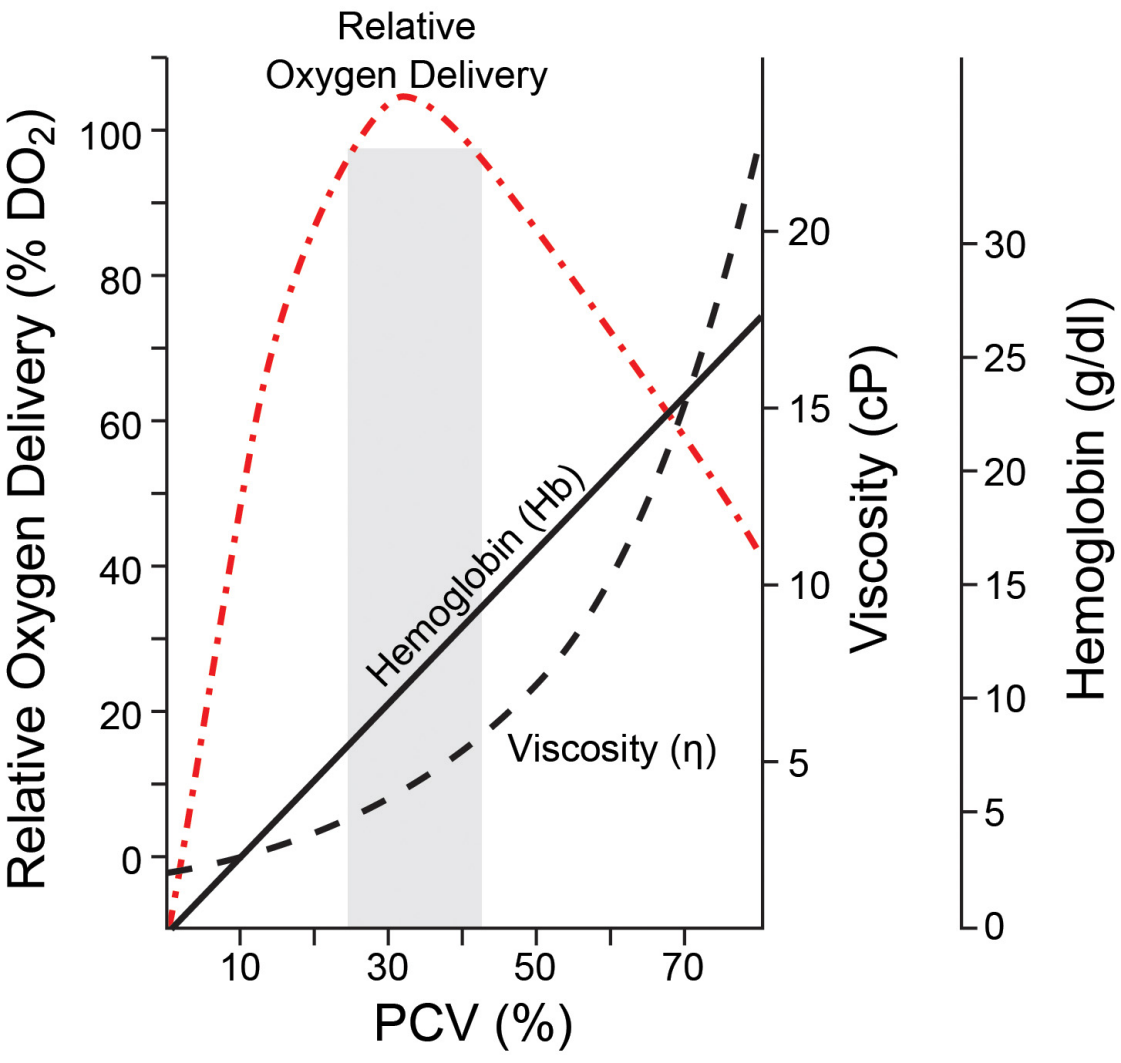
Relative oxygen delivery. The effects of packed cell volume (PCV), hemoglobin concentration (g/dl), and blood viscosity (η) on relative oxygen delivery (% DO_2_). Relative oxygen delivery is maximized at a hemoglobin concentration of ≈12 g dl^−1^ and blood viscosity of approximately 6–9 cP. Deviations in PCV, hemoglobin concentration, and blood viscosity from optimal values result in decreases in relative oxygen delivery.

The majority of oxygen delivered to tissues in vertebrates is carried by hemoglobin (e.g., 1.34 ml O_2_/gm Hb) within red blood cells (e.g., RBCs). The percentage of RBCs in blood is the hematocrit (e.g., Hct: ϕ). Increases in Hct increase blood η and arterial blood oxygen content (e.g., CaO_2_), but decrease vascular conductance, Q, and therefore DO_2_ (Figure 7). Optimal hematocrit theory posits that there is an optimal ϕ and ϕ/η that maximizes tissue oxygen supply (J_ox_) (21, 124, 125). Optimal ϕ values in vertebrates inform transfusion triggers and personalized transfusion strategies (126, 127). Changes in η that promote Q (Equation 5): η becomes less sensitive to Hct in smaller blood vessels (e.g., < 500 and >10 μm) due to RBC central streaming (e.g., Fahraeus–Lindqvist effect) (128). The Fahraeus–Lindqvist effect, however, has minimal effect on η in capillaries (e.g., blood vessels < 10 μm) since RBCs travel through capillary vascular beds consecutively and RBC diameter can be greater than the capillary diameter (21, 129). Maximum DO_2_ therefore becomes dependent upon the product of capillary blood flow (Q_c_) and CaO_2_ (Equations 15–17):


$$\begin{array}{l} \text{Ca}{\text{O}}_{\text{2}}=\;\left(\text{Hb x 1}.\text{34 x Sa}{\text{O}}_{\text{2}}\right)\;+\text{Pa}{\text{O}}_{\text{2}}\text{x}0.00\text{3}) \end{array}$$ (15)

$$\begin{array}{l} {\text{Q}}_{\text{c}}=\text{ΩΔPc}/\;\eta \;\left(\;\varphi \;\right) \end{array}$$ (16)

$$\begin{array}{l} \text{D}{\text{O}}_{\text{2}}=\;{\text{Q}}_{\text{c}}\text{x Ca}{\text{O}}_{\text{2}} \end{array}$$ (17)

where Hb is the hemoglobin concentration (g/dl), 1.34 is the amount of ml of O_2_ bound to each gram of Hb, SaO_2_ is the percent oxygen saturation of Hb, PaO_2_ is the partial pressure of oxygen in blood (mm Hg), 0.003 is the ml of O_2_ dissolved per mm Hg of PaO_2_, Ω is a constant dependent on capillary geometry, Pc is the capillary pressure change, and η (ϕ) represents the dependence of η on hematocrit (ϕ). Substituting for Q_c_ from Equations 16, 17 gives:


$$\begin{array}{l} {\text{J}}_{\text{ox}}=\text{b}\Omega \text{ΔPc}\left[\;\phi \;/\;\eta \;\left(\;\phi \;\right)\right]\text{xCa}{\text{O}}_{\text{2}} \end{array}$$ (18)

where J_ox_ is oxygen supply, and b is a proportionality constant (21). Tissue oxygen supply (J_ox_) depends on microvascular CaO_2_, Pc, Q_c_, ϕ, and η (Equations 15 and 18). The CaO_2_ and J_ox_ equations, however, do not account for acid environments (e.g., acidemia). Acidemia causes a decrease in Hb affinity for O_2_ (i.e., right shift in the O_2_-Hb dissociation curve), making O_2_ more available but also lowering CaO_2_ (e.g., a 0.1 decrease in pH decreases SaO2 by 10%−15%) and O_2_ transport (127, 130).

Intravenous fluid administration (i.e., crystalloid or colloid) can improve Q_c_, DO_2_, and PtO_2_ based on enhancements in Pc, ϕ, and η or can decrease Q_c_, DO_2_ and PtO_2_ because of acute dilution of ϕ by 30% or more (e.g., Hb ≤ 8–10 g/dl), tissue edema-induced obstruction of Q_c_, or increases in intercapillary distance (131). Notably, neither crystalloids nor colloids provide adequate quantities of oxygen to support tissue viability, even when exposed to elevated oxygen concentrations (e.g., 100%) (132). The amount of O_2_ physically dissolved in solution (e.g., 1.8 ml/dl: 0.003 × 600 mm Hg) when breathing 100% oxygen at sea level is well below the minimum tolerable tissue oxygen requirement (DO_2crit_) for most tissues (e.g., < 5–7 ml/kg/min) (133). The potential benefits of hyperbaric oxygen therapy remain unresolved (134).

## Interstitium and lymphatic system

**Figure 8:**
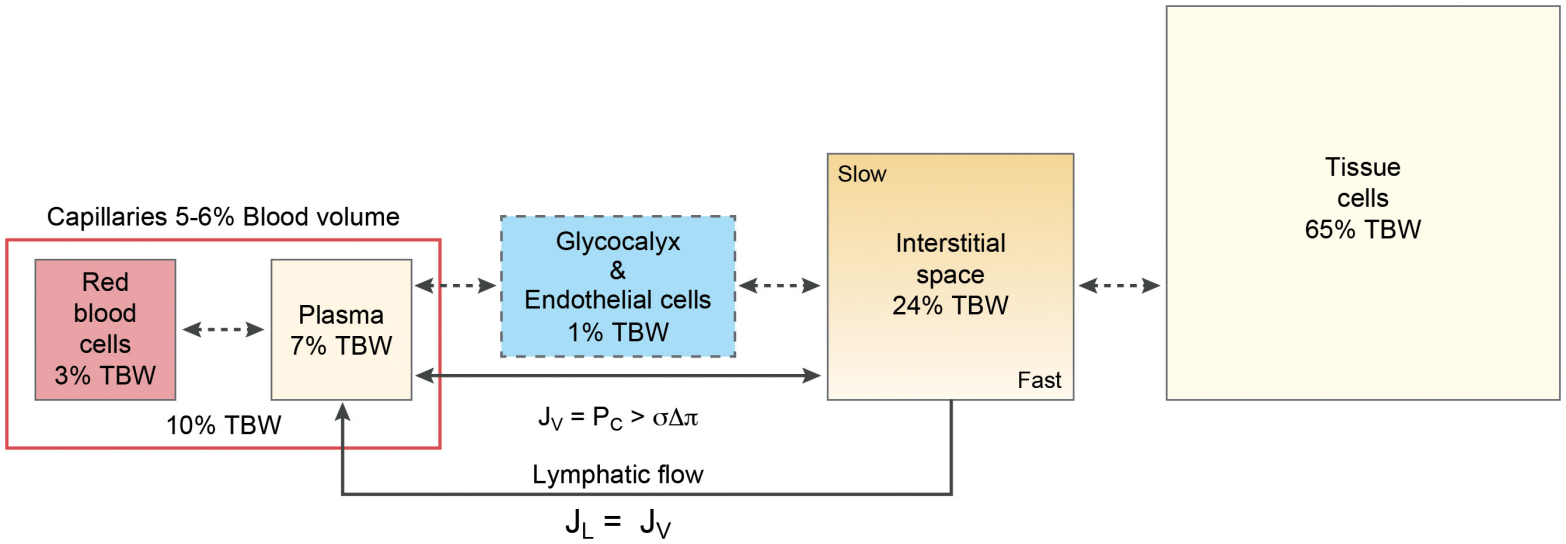
Body water distribution as a percentage (%) of total body water (TBW). Body fluid balance is maintained when lymphatic flow (J_L_) equals transcapillary fluid flux (J_V_). Pc is the primary determinant of J_v_ and is greater than σ*Δπ* during steady-state conditions.

The interstitium and lymphatic system are vital components of fluid balance and circulation (Figure 2) (135, 136). The interstitial space is a contiguous, dynamic fluid compartment and reservoir composed of both loose and dense connective tissue and fascia (e.g., fibrous network and gel) that provide structural support for interstitial components (e.g., nerves, blood and lymphatic vessels, and cells) while acting as a mechano-transduction zone that orchestrates immune responses and maintains cell viability (Figure 8) (137–139). Interstitial fluid volume is regulated by dynamic adjustments in capillary hydrostatic and oncotic forces, volume-dependent changes in interstitial tissue capacitance, compliance, and conductance, and lymphatic pumping (136, 137). Integrins act as essential mechanoreceptors that modulate interstitial physical properties and Pi (138). The lymphatic system is a ubiquitous, one-way, dynamic network of blind-end vessels that passively and actively return interstitial fluid (e.g., filtered plasma and lymph) to the venous circulation, thereby helping to maintain a negative interstitial pressure (140). Lymph is transferred by systemic lymph vessels to lymph nodes and then to efferent lymphatic vessels that empty into the thoracic or right lymphatic ducts (141–143). Interstitial pressure (Pi), one-way valves, intrinsic lymph vessel contractions (e.g., lymph vessel smooth muscle), and extrinsic factors (e.g., skeletal muscle contraction, arterial pulsations, and breathing-associated changes in intrathoracic pressure) compress lymph vessels to facilitate lymph flow (144–147). Pseudo-lymphatic (i.e., glymphatic) “channels” and meningeal lymphatic vessels drain cerebrospinal fluid into a cervical glymphatic drainage system that empties into cervical subclavian veins (148). The glymphatic system is key to the maintenance of intracranial pressure by returning cerebrospinal fluid entering from perivascular spaces and capillary filtration to meningeal lymphatics that drain into cervical lymph nodes (149–151).

### Interstitium

**Figure 9:**
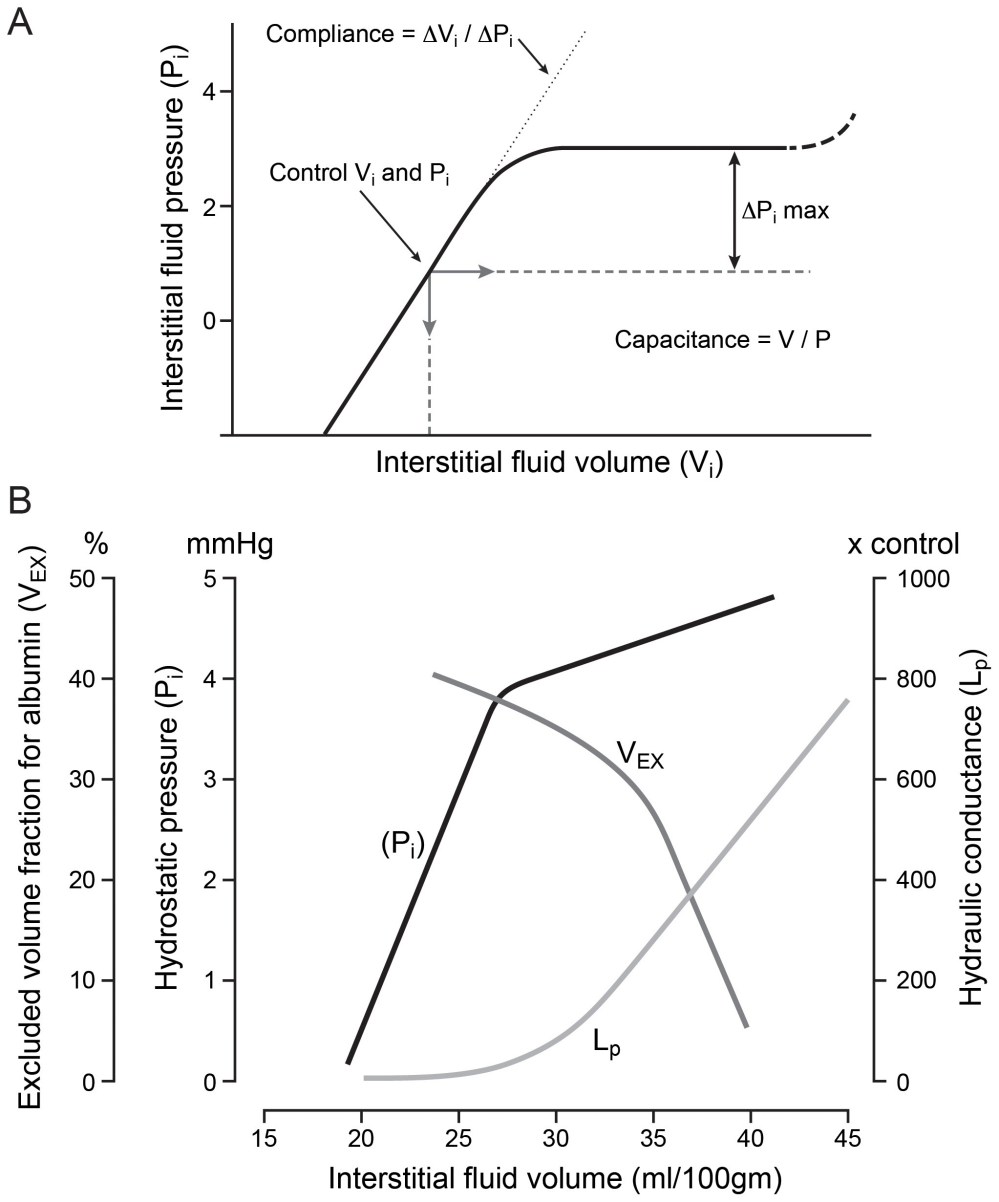
Tissue compliance, capacitance, and excluded volume. **(A)** Changes in interstitial compliance (ΔV/ΔP) and capacitance (V/P) associated with increases in interstitial fluid volume (V_i_). Interstitial compliance and capacitance markedly increase once interstitial pressure (Pi) increases +2 mm Hg. **(B)** Changes in interstitial pressure (Pi), hydraulic conductance (L_p_), and excluded volume (V_EX_) for albumin result from increases in interstitial fluid volume (modified from reference (154)).

Interstitial tissue is a hybrid structure that is 90%−95% water and contains 20%−25% of total body water by weight, along with cells (e.g., fibroblasts, macrophages, and lymphocytes) and an entangled network of locally produced proteins (collagens, elastin), proteoglycans (e.g., fibronectin), and polysaccharides (e.g., glycosaminoglycans: GAGs) that surround nerves, blood vessels, and most parenchymal cells. Interstitial tissue is most prevalent in subcutaneous and connective tissue linings, the gastrointestinal tract, surrounding muscle cells and blood vessels, and the lungs (138). GAGs are negatively charged polysaccharides (e.g., chondroitin sulfate, heparan sulfate, and hyaluronic acid) that attract sodium ions and create an osmotic gradient that binds water (137, 138). Water binding to collagen and GAGs produces a spongy, expandable, reticular environment (i.e., extracellular matrix, ECM) that inhibits the dispersion of larger molecules (e.g., albumin) and creates an interstitial excluded volume (V_Ex_) (152–154). The collagen fiber network and integrin–collagen binding restrain the natural tendency for GAGs to expand, creating a negative pressure (i.e., pulling force) that plays a pivotal role in determining interstitial compliance and capacitance (Figure 9A) and Pi, Lp, and V_ex_ (Figure 9B) (145, 155–157). Approximately 99% of interstitial water is bound to GAGs and collagen under normal conditions, with the remainder being unbound (e.g., < 1%) or free water (140). Interstitial solute accessibility determines fluid balance, osmotic pressure, and fluid transport efficiency, while interfacial nano-scale fluid transport zones hold and move free fluid along cutaneous, perivenous, periarterial, and perineural channels (e.g., microtubular fibrorail) toward lymphatic vessels (158, 159).

Interstitial compliance (2–3 ml/mm Hg/kg) and pressure (−1 to −2 mm Hg) are relatively stable in healthy tissues but change dramatically during interstitial inflammation or fluid accumulation (Figure 9A) (140, 160, 161). Increases in interstitial fluid volume (e.g., Vi) modify interstitial stress relaxation (i.e., compliance), solute accessibility, and IV fluid disposition (i.e., distribution and elimination) (162). Physiologically, total interstitial fluid volume (e.g., V_i_) can be divided into two reciprocally related volumes: excluded (e.g., V_Ex_) and accessible (e.g., V_Acc_), based on the size-dependent exclusion or accessibility of large molecules (V_i_ = V_Ex_ + V_Acc_). The V_Ex_ is the dynamic, hydration-dependent (e.g., greater hydration less exclusion) portion of the interstitium generated by large, negatively charged indigenous molecules (e.g., collagen, proteoglycans, GAGs) (138, 163, 164). These large indigenous molecules (i.e., macromolecular crowding agents) exclude smaller negatively charged water-soluble molecules like albumin, hyaluronan, and immunoglobulins from occupying the same space, thereby reducing the space available for macromolecular distribution (163–165). The V_Ex_ is denser and less permeable, compliant, and accessible compared to the V_Acc_, acting as a physical barrier to large macromolecules (e.g., albumin and IgG proteins) and immune cells (166). The interstitial accessible volume (V_Acc_) refers to the space into which specific macromolecules (e.g., albumin) can distribute. The V_Acc_ contains bound and free water. The V_Ex_ decreases the total interstitial fluid space for the diffusion of albumin by approximately 40%−50%, thereby forcing it to equilibrate into a smaller portion of the interstitial volume (e.g., V_Acc_ for albumin). The potential ambiguity between “1% free fluid” and V_Acc_ is resolved by distinguishing between the physical state of water (e.g., free vs. bound) and the space available for molecular transport. Solutes like glucose, electrolytes, and smaller proteins diffuse more freely through the ECM than macromolecules, even if most of the water is bound (166). Fluid flow in the ECM is not uniform, however, and is greatly dependent on the local architecture and density of the surrounding ECM components (167).

Increases in Vi expand GAGs, decreasing GAG density and transitioning macromolecular exclusion from steric to charge effects (166). The fractional excluded volume (V_Ex_) for albumin may increase due to GAG expansion and electrostatic repulsion; however, the albumin V_Acc_ eventually increases because V_i_ increases much more than V_Ex_ and the concentration of macromolecular crowding molecules decreases (154). These changes are responsible for the development of the non-linear portion of the interstitial pressure-volume relationship (Figure 9A) (166).

### Lymphatic system

The lymphatic system maintains interstitial fluid balance and is crucial for total body fluid circulation. Increases in intravascular volume and transcapillary fluid flux (Jv) accelerate lymphatic flow and the return of fluid (i.e., lymph), proteins (e.g., albumin), and fats (e.g., chyle) to the bloodstream, helping to maintain or restore intravascular volume (168, 169). Increases in Vi to values greater than 20% (>30 ml/kg increase in V_i_) of its resting volume decrease GAG density and V_Ex_ and increase V_Acc_, interstitial C_com_, C_cap_, and G due to the fracture of integrin–collagen and integrin–fibronectin (i.e., molecular bridges) cross-links (163, 164). Notably, interstitial overhydration can “wash out” interstitial hyaluronan, making it more difficult for the interstitium to return to its original conformation and volume (165, 168, 169).

Early fluid replacement studies conducted in human surgical patients and dogs subjected to hemorrhagic shock suggested that the extracellular fluid compartment contracts during blood loss and that IV fluids become sequestered in a compartment that is “not available” for circulation, an “unknown third space” (170–173). The magnitude of third space fluid loss was correlated with the severity of injury and the amount of fluid administered (174). An anatomical location for the lost fluid was not initially identified, although it was theorized that fluid could have accumulated inside cells, the intestinal tract, or traumatized tissues (174, 175). Subsequent research established that these earlier studies were based on “flawed methodology” and that “fluid accumulates interstitially” (176–179). Cumulative increases in Vi eventually decrease integrin collagen-binding to GAGs and activate mechano-transduction pathways that release matrix metalloproteinases (e.g., MMPs), resulting in GAG disentanglement (i.e., degeneration), a decrease in GAG electrostatic water binding, and increases in gel-free water that elevate interstitial C_cap_, C_com_, and G (153, 180, 181).

Maintaining microvascular–interstitial–lymphatic integrity and interactions is vitally important for preserving interstitial fluid balance (19, 182). Lymph flow (J_L_) increases to match transcapillary fluid flux (Jv) and is directly dependent upon P_i_, intrinsic lymphatic pumping pressure (P_lp_), external compression (e.g., muscle contraction), and venous pressure (Pv), and is inversely related to the effective lymphatic resistance (R_L_; Equation 19).


$$\begin{array}{l} {\text{J}}_{\text{L}}=\;{\text{P}}_{\text{i}}+\;{\text{P}}_{\text{lp}}-\;{\text{P}}_{\text{v}}/{\text{R}}_{\text{L}} \end{array}$$ (19)

Increased Jv increases Vi, Pi, P_lp_, and J_L_. Excess Vi or reductions in J_L_ lead to Vi-associated increases in C_cap_, C_com_, and G.

## Fluid retention, reabsorption, and redistribution

Fluid retention, reabsorption, and redistribution are physiological mechanisms for maintaining and restoring the effective circulatory volume (ECV): the portion of the extracellular fluid in the arterial system required to adequately perfuse tissues (183). Failure in any single component can lead to fluid imbalance, interstitial fluid accumulation, and the potential for decreased tissue nutrient delivery. Increased sympathetic activity in response to stress, fear, pain, hypovolemia, or hypotension can trigger fluid retention, reabsorption, and redistribution (184, 185). Decreased baroreceptor activity due to hypotension or hypovolemia increases central nervous system sympathetic activity, decreases parasympathetic outflow, activates the renin–angiotensin–aldosterone system (e.g., RAAS), and promotes the release of antidiuretic hormone (i.e., vasopressin) from the posterior pituitary gland (186). Increased sympathetic activity also relaxes the bladder and constricts the urethral sphincter (187). These physiologic adaptations are particularly relevant since the prevalence of fluid overload is estimated to range from 10 to 35% or higher in human critical care patients (180, 188–190). The incidence of fluid overload in animals is underreported.

### Fluid retention

Fluid retention predisposes to extracellular (e.g., interstitial) fluid accumulation when transcapillary fluid flux (e.g., J_v_) exceeds lymphatic drainage (J_L_) (191–193). General anesthesia, anesthetic drugs (e.g., inhalant anesthetics, opioids, and non-steroidal anti-inflammatory drugs), mechanical ventilation, and long-term IV fluid administration promote interstitial fluid accumulation by increasing membrane fluidity (e.g., permeability) (194–197) and impairing renal tubular function (198–201) or lymphatic pump function (Box 2) (104, 202, 203). Opioids and benzodiazepines increase ADH secretion, and non-steroidal anti-inflammatory drugs inhibit the production of vasodilating prostaglandins, reducing renal tubular fluid reabsorption and glomerular filtration, respectively (204–206). A 4-h study in anesthetized dogs administered oxymorphone, atropine, propofol, isoflurane, with or without carprofen, and 10 ml/kg/h lactated Ringer's solution reported urine output below normal values (e.g., 0.46 ml/kg/h; 1–2 ml/kg/h) (207). This same study also reported an increase in total body water and extracellular fluid volume (207). Other studies have demonstrated that inhalant anesthetics alone stimulate the renal sympathetic nervous system more than intravenous propofol anesthesia, resulting in decreased renal perfusion, oxygenation, and water and sodium excretion (208, 209). Mechanical ventilation can cause fluid retention by reducing cardiac output, triggering ADH release, inhibiting atrial natriuretic peptide (ANP) release, and impairing lymphatic drainage (210, 211).

Box 2 Factors that promote interstitial fluid accumulation and edema.

1. Excessive IV fluid administration
  a. Loss of the interstitial excluded volume (e.g., overhydration)
2. Altered Starling forces
  a. Increased capillary hydrostatic pressure
  b. Decreased capillary oncotic pressure
  c. Decreased interstitial hydrostatic pressure
  d. Increased interstitial oncotic pressure
3. Increased capillary permeability (e.g., endotheliopathy)
4. Decreased lymphatic pumping or obstruction
  a. Anesthetic drugs
  b. NSAIDS
  c. Corticosteroids
  d. Calcium channel blockers
  e. Long-term fluid administration
5. Extracellular matrix disruption (e.g., inflammation and allergic reactions)
6. Disease
  a. Heart and renal failure (e.g., Na^+^ and H_2_O retention)
  b. Liver failure (e.g., reduced albumin)
7. Traumatic events (e.g., tissue injury and burns)
8. Autonomic dysfunction (i.e., dysautonomia)
9. Gravity
10. High altitude (e.g., hypoxic pulmonary vasoconstriction)

### Reabsorption (transcapillary refill)

Transcapillary refill is normally dependent on acute decreases in Pc or increases in π_c_ (113). Interstitial fluid reabsorption is rapid, transient, and depends on the magnitude of the Pc pressure decrease, the plasma–interstitial osmolarity difference, and the subject's state of hydration (113). Transcapillary refill from interstitial splanchnic organs, skeletal muscle, and skin begins immediately after mild (e.g., 5–15 ml/kg) to moderate (e.g., 15–35 ml/kg) blood loss and can restore 60%−80% of lost plasma volume within 60–90 min (212). Interstitial fluid reabsorption from the splanchnic viscera (e.g., fenestrated and sinusoidal capillaries) and skeletal muscle (e.g., continuous capillary; large mass) is likely the primary source of fluid reabsorption due to their large tissue mass. The previously described no reabsorption rule does not prevent transcapillary refill from transiently occurring in non-fenestrated capillaries or from fenestrated (e.g., kidneys, small intestine, and endocrine glands) or sinusoidal capillaries (e.g., liver) following hypovolemic and hypotensive (i.e., dynamic) events or in response to marked increases in plasma osmolarity (e.g., hypertonic glucose solutions; hypertonic saline) (109, 113).

Sympathetic nervous system activation facilitates interstitial fluid reabsorption (i.e., transcapillary refill) by producing arteriolar vasoconstriction (e.g., alpha-1 effect), thereby lowering P_c_ and increasing the capillary surface area for fluid absorption (e.g., beta-2 effect) (113, 213). Application of this knowledge to clinical practice, however, is complicated by changing patterns of Pa and P_V_ induced by local microcirculatory endogenous (e.g., nitric oxide and prostacyclin) regulators, and the confounding effects produced by the co-administration of vasoconstrictive (e.g., alpha-1 and alpha-2) or vasodilating drugs (e.g., sedatives, anesthetics, and beta-2 agonists) (106). Similarly, the administration of vasoconstrictors (e.g., norepinephrine and phenylephrine) or vasodilators (e.g., nitroprusside and nitroglycerin) can either increase or decrease P_C_ depending on the pathophysiologic processes operating at the time of drug administration (214–217).

### Redistribution

Acute blood loss (10%−15% blood volume), hypotension (e.g., < 60 mm Hg; 20% decrease in MAP), and hypoxemia (e.g., PaO_2_ < 60–70 mm Hg; SpO_2_ < 95%) reduce DO_2_ and initiate neurohumoral mechanisms, predominantly sympathetic, that increase cardiac performance and modulate vascular tone prioritizing blood flow distribution to the tissues that need it most (218). Redistribution of blood volume from the body's blood reservoirs (e.g., splanchnic viscera, liver, and spleen) increases the ECV and red blood cell mass (e.g., splenic contraction), improving tissue perfusion and oxygenation. Veins contain approximately 70% of the total blood volume, and small veins and venules contain approximately 45–55% of the total venous blood volume (27). Sympathetically mediated blood volume redistribution is triggered within seconds to minutes following euvolemic or hypovolemic hypotensive events (219–222). Splanchnic veins (e.g., spleen, liver, and intestines) have a large capacitance (e.g., 20%−30% blood volume), are more than 30 times more compliant than arteries (e.g., arteries: 0.07 ml/kg/mm Hg; veins: 2.5 ml/kg/mm Hg; and splanchnic veins: 2.8 ± 0.6 ml/kg/mm Hg), and contain up to five times the density of adrenergic receptors compared to arteries (223–225). Splanchnic veins can contribute (i.e., redistribute) between 8 and 15 ml/kg of blood to the ECV (223–225). Controlled hemorrhage experiments in healthy humans and animals suggest that a 10%−15% loss of blood can be tolerated without significant changes in blood pressure, heart rate, or central venous pressure because of blood redistribution (226–228). Larger volumes of blood loss (e.g., >15%) trigger venous and arterial vasoconstriction (224). Notably, activation of the sympathetic nervous system also increases circulating red blood cell mass in most animals to a far greater extent than observed in humans (e.g., humans ≈5%; cats ≈20%; dogs ≈30%; and horses >50%) (229, 230). For example, the adult horse's spleen can store up to 20% of its total blood volume, contains up to 30% of the red blood cell mass, and can redistribute 50%−70% of its volume to the ECV during intense activity, stress, excitement, and blood loss (231–233).

## Intracellular fluid volume

**Figure 10:**
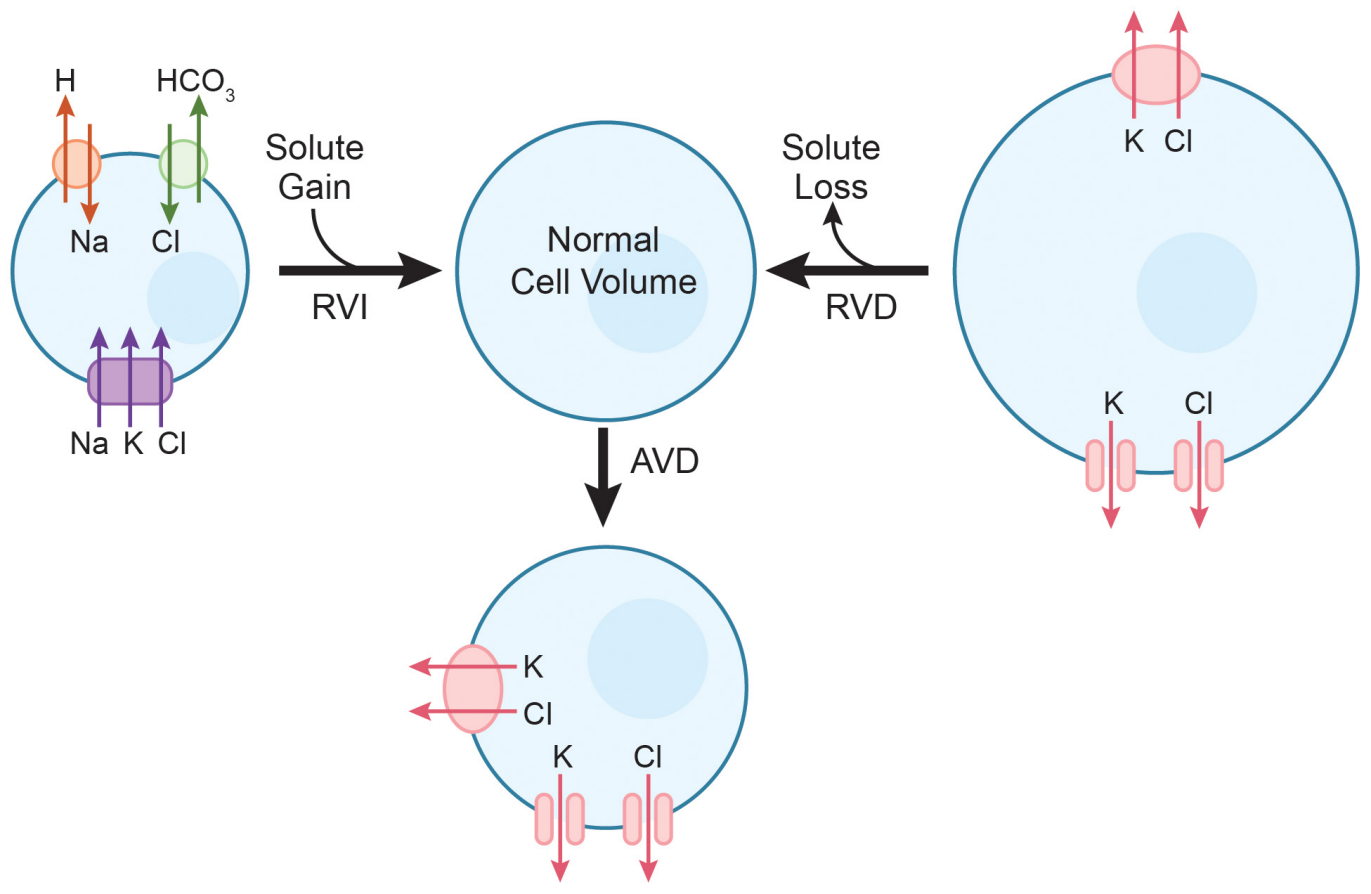
Regulatory cell volume changes. Regulatory cell volume increases (RVI) and decreases (RVD) are determined by intracellular or extracellular solute concentrations that generate transmembrane osmotic gradients (π) and are primarily mediated by extracellular Na^+^ concentration and the production of intracellular osmolytes. Decreased tissue oxygen and low tissue pH impair mitochondrial function, leading to ATP depletion, reactive oxygen species (ROS), and ion imbalances (Na^+^ influx, K^+^/Cl^−^ efflux) that trigger apoptotic volume decrease (AVD).

Cell volume is a critical determinant of cell metabolism, proliferation, migration, and death and is directly dependent upon tissue hydration, interstitial tonicity, and the continuous exchange of nutrients (e.g., DO_2_) for waste products (234). It is presumed that hypertonic fluids decrease intracellular fluid volume, isotonic fluids maintain intracellular fluid volume, and hypotonic fluids increase intracellular volume. Water and small solutes (e.g., sodium and chloride) move relatively freely across capillary walls into the interstitial space, diluting interstitial proteins and lowering π_i_, and are expected to increase cell volume. Cells, however, can regulate their own volume during modest changes in extracellular osmotic conditions via mechanosensitive water channels (e.g., aquaporin), changes (e.g., seconds) in membrane ion pump activity, and cell membrane ion exchange mechanisms—processes collectively referred to as regulatory volume increase (RVI) or decrease (RVD; Figure 10) (234, 235). The cell membrane ion transporters and channels involved in RVD and RVI have been identified (234, 236, 237), and any gain or loss in cell water is immediately sensed and corrected (234, 238). Isotonic solutions (e.g., 0.9% normal saline, Lactated Ringer's) have an osmolality similar to plasma and are expected to cause minimal cellular fluid shifts or changes in intracellular volume in normal animals. Studies in conscious healthy human volunteers, as well as surgical and critically ill patients, however, indicate that larger volumes of IV isotonic fluids can reduce intracellular water and significantly increase extracellular water (239, 240). Large fluid infusions also produce a decrease in microcirculatory total and perfused vessel density (e.g., TVD and PVD) that persists for several days after macrocirculatory parameters (e.g., CO, MAP) return to normal (241). These responses require further investigation but could be caused by cell stress (e.g., hypoperfusion, hypoxia, hypercarbia, and acidosis) induced apoptotic volume decrease (AVD; Figure 10) (235, 242–244). Cell stress-induced AVD helps to prevent cell degradation and the release of harmful cellular components (e.g., lactic acid, reactive oxygen species, and cytokines) into the interstitium (244).

## Pharmacokinetics

Pharmacokinetic (PK) studies are conducted to determine drug behavior in the body. Compartmental, non-compartmental, and physiologically based PK (PBPK) methods utilize blood or plasma drug concentration–time data to obtain statistically derived parameters that quantitatively describe drug absorption, distribution, metabolism, and excretion (245, 246). Drugs are administered into, dispersed, and eliminated from a hypothetical central compartment or volume (Vc). A pharmacokinetic compartment (e.g., Vc) is an imaginary, mathematical, or computer-generated space (i.e., not anatomical or physiological), or a space within another space (245). A compartment is not considered a real space (e.g., ECFV) unless the drug's physicochemical properties (e.g., lipid solubility, protein binding, molecular size, and charge), distribution, and elimination can be accurately accounted for. For example, estimation of Vc from concentration–time data of an IV solute that purportedly stays in the plasma (e.g., Evans blue dye) almost always provides a larger volume than the actual PV because of some distribution into highly perfused tissues (247). Pharmacologic compartmental models provide insight into drug disposition (e.g., distribution or elimination) and are used to determine drug dose, dosage, and simulate outcomes for different drug dosing schedules (230). Physiologically based pharmacokinetic models are more biologically realistic than PK models because they incorporate factual physiological, anatomical, and biochemical data; however, they are difficult to perform since they require complex measurements of physiologic parameters (P, Q, R, G, Jv, etc.) that may be difficult to obtain or validate (248).

The number of compartments required to describe a drug's behavior is determined by sampling and plotting drug plasma concentration–time data for as long as the drug can be accurately detected. The concentration–time data profile determines the model (i.e., the model is fitted to the data). Zero- and first-order kinetics describe the drug's rate of elimination from the central compartment (i.e., Vc). Zero-order kinetic drugs are eliminated from Vc at a constant rate, while first-order kinetic drugs are eliminated at a rate that is directly proportional to the amount (i.e., concentration) of drug remaining in the compartment from which it is being eliminated (Equation 20).


$$\begin{array}{l} {\text{X}}_{\text{t}}=\;{\text{X}}_{0}\text{ x }{\text{e}}^{-\text{kt}} \end{array}$$ (20)

X_t_ is the amount of drug in the body at time t, X_0_ is the amount of drug administered at time zero, e is the Euler number (i.e., the constant that is the base for natural logarithms), k is the rate constant expressed as the reciprocal of time, and t is the elapsed time since the drug was administered. The negative sign (–) indicates that the drug concentration at time zero (e.g., X_0_) is decreasing (i.e., undergoing exponential decay) because X_0_ is decreasing (i.e., being eliminated). The elimination rate for most fluids administered into Vc is described by first-order kinetics and represented by one-, two-, or three-compartment models (245, 249–253).

### Volume kinetics

**Figure 11:**
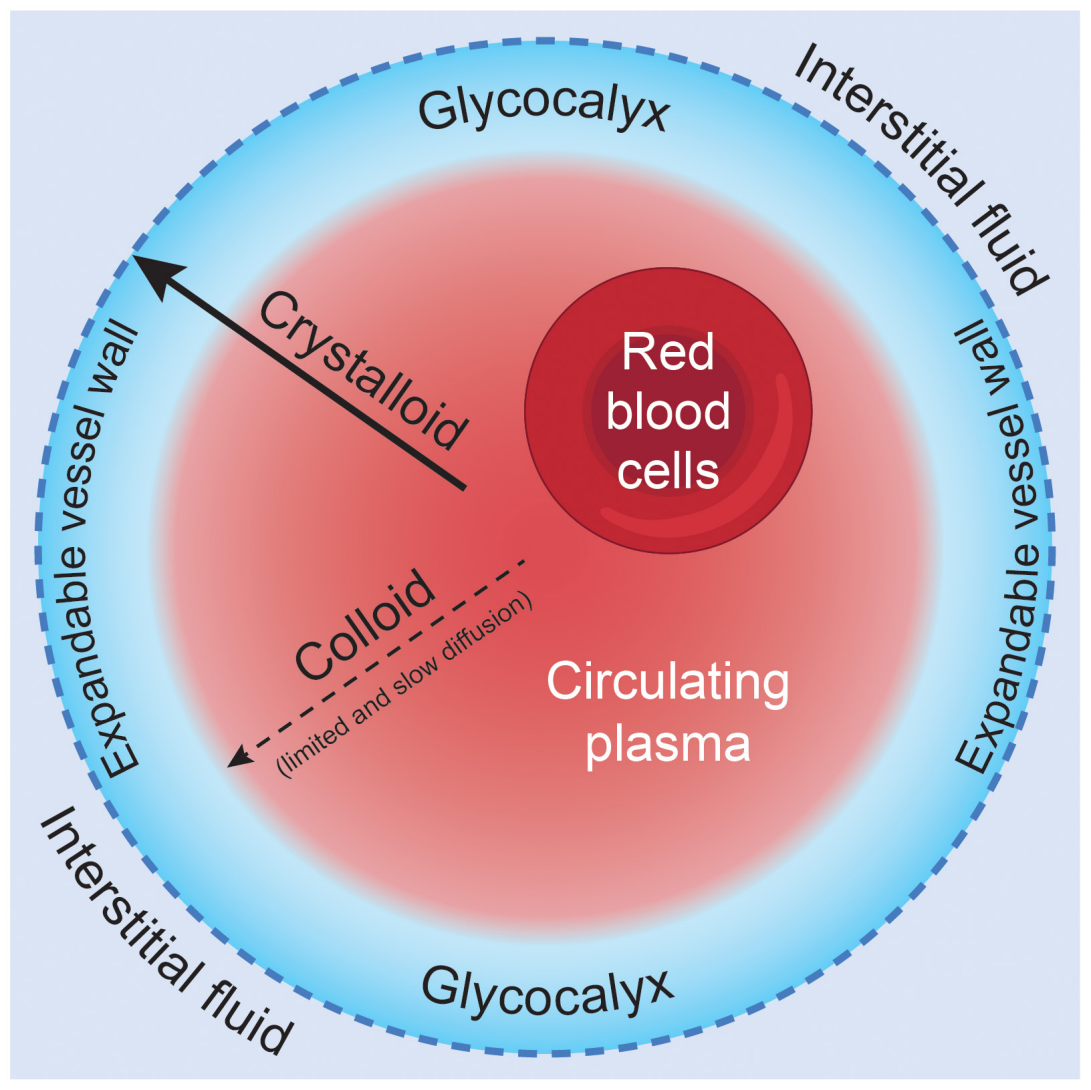
Crystalloids and colloids. Colloid administration results in greater volume expansion than crystalloid administration. The molecular size of colloids limits their distribution within Vc and markedly delays convection and diffusive solute flux (Js: transcellular pathway) from the plasma to the interstitium.

Volume kinetics utilizes PK principles to describe, predict, and prescribe intravenous fluid therapy (Table 1) (249–254). VK analysis measures the plasma dilution (P_D_) of an easily identifiable biological plasma analyte (e.g., S: hemoglobin [Hb] or albumin [Alb]) to estimate changes in plasma volume (e.g., PV) since the concentration of a fluid cannot be directly measured (Equation 21):


$$\begin{array}{l} {\text{P}}_{\text{D}}(\text{t})\;=\;\left[\text{H}{\text{b}}_{0}/\text{H}{\text{b}}_{\text{t}}-\text{1}\right]/\text{1}-\text{Hct }=\;[\text{PV}(\text{t})\\ \;-\text{PV}\left(0)\right]/\text{PV}(0) \end{array}$$ (21)

where P_D_ (t) is plasma dilution at any time t during IV fluid infusion, Hb_0_ is hemoglobin concentration at t = 0 before intravenous infusion (g/dl), Hb_t_ is hemoglobin concentration at any time t after intravenous infusion (g/dl), Hct (0) is hematocrit at t = 0 before intravenous infusion, PV(0) is plasma volume at any time t before intravenous infusion (ml), and PV(t) is plasma volume at any time t after intravenous fluid infusion (ml). Data obtained by measuring the dilution of a stable water-soluble analyte (e.g., Hb or Alb) is inversely related to the increase in plasma water. Greater analyte dilution indicates a larger volume produced by the infused fluid. The extrapolated volume changes during the initial distribution phase to time zero provides a reasonable estimate of the subject's plasma volume, even though compartmental spaces are considered theoretical. The total amount of the measured plasma analyte (e.g., Hb or Alb) is assumed to remain unchanged throughout data collection to accurately determine changes (e.g., dilution) in plasma analyte concentration (249, 254). For example, increases in blood Hb concentration caused by splenic contraction during fluid administration would increase Hb_t_ and decrease PV (t), while Hb sequestration (e.g., splenic entrapment) would lower Hb_t_ and be erroneously interpreted as an increase in PV(t). The Vc for IV fluids (e.g., crystalloids and colloids) always includes the plasma volume but is often determined to be larger than the actual plasma volume following crystalloid administration due to their rapid penetration of the GCX and distribution out of the plasma into surrounding tissues (252–254). Crystalloids (e.g., normal saline and lactated Ringer's solution) rapidly penetrate the GCX, while colloids are initially confined to a volume within the GCX, resulting in greater plasma dilution (e.g., Hb or Alb) and a more sustained (e.g., longer half-life) increase in circulating plasma volume (Figure 11) (83, 255–257).

**Table 1:** Volume kinetics compartmental model parameters.

Parameter	Unit	Description
Fluid infusion rate: (R_0_, k_1_)	ml/kg/min ml/kg/h	Constant rate infusion (CRI)
Compartments: V_c_ V_P_ (V_t_, V_t1_), V_t1_ (V_F_) V_t2_ (V_S_)	ml/kg	One-compartment fluid space: 1-VOFC Two-compartment fluid space: 2-VOFS Three-compartment fluid space: 3-VOFS V_S_ represents an overflow reservoir or “third space”
Central compartment (V_c_)	ml ml/kg	Immediate volume into which the fluid is distributed: approximates PV
Volume of distribution (Vd)	ml ml/kg	Apparent volume the fluid has distributed into at time (t); The Vd at time (t) zero approximates PV
Clearance (Cl)	ml/min	Rate of fluid removal from the central compartment:
k_12_	min^−1^	Rate of fluid distribution into V_p_, V_2_, V_t1_
k_21_	min^−1^	Rate of re-distribution of fluid out of V_p_, V_2_, V_t1_
k_23_ and k_32_	min^−1^	Rate of fluid movement into (k_23_) and out of (k_32_) V_t2_
k_10_	min^−1^	Rate of fluid eliminated by renal excretion (urinary output)
k_b_	min^−1^	Represents elimination rate constant for non-renal basal fluid losses from the central fluid space
Elimination half-life (t1/2)	min, h	The time it takes for the plasma volume expansion (the excess fluid in the blood) to decrease by half

Volume of fluid space (VOFS); PV, plasma volume; Vc PV; VP = Vt, V_t1_; V_t1_ = V_F_; V_t2_ = VS; 2-VOFS model: k_12_ and k_21_ the rate of fluid movement between Vc and VP (V_t_, V_t1_); 3-VOFS model: k_23_ and k_32_ describe the rate of fluid movement between fast (V_t1_, V_F_) and slow equilibrating spaces (V_t2_, V_S_).

**Figure 12:**
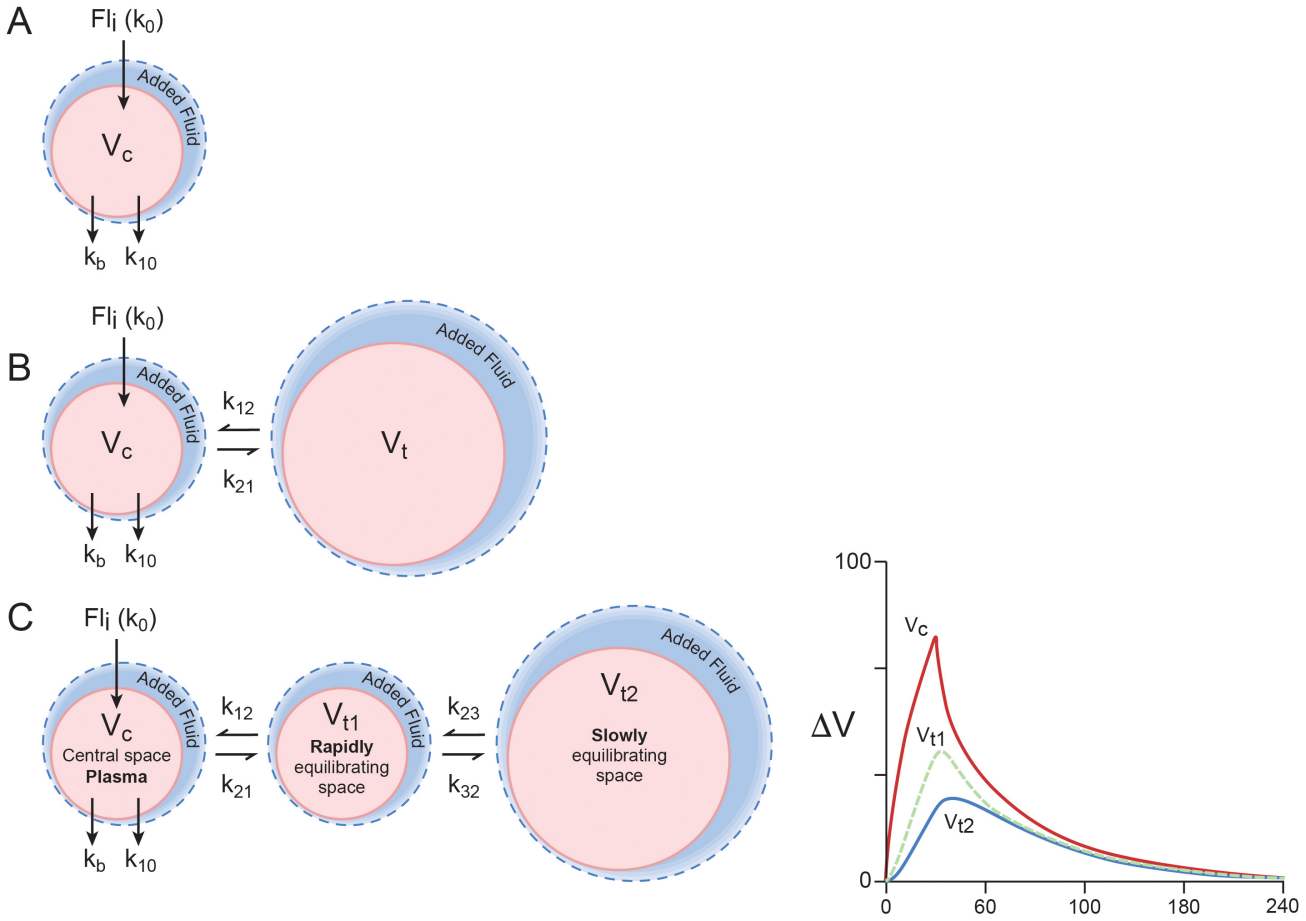
One, two, and three compartment volume kinetic models. **(A)** One compartment model: Fluid is infused (Fl_i_) at a constant rate (k_0_) into an expandable (e.g., added fluid) central compartment (V_c_) from which it is eliminated (k_b_, k_10_). k_b_ represents non-renal water losses (e.g., respiration, sweating, and feces) and k10 represents urinary excretion. **(B)** Two compartment model: Fluid is infused (Fl_i_) at a constant rate (k_0_) into an expandable (e.g., added fluid) central compartment (V_c_) and transferred (k_12_) from V_c_ to a peripheral compartment (V_t_; added fluid) and from V_t_ back to V_c_ (k_21_). **(C)** Three compartment model: Fluid is infused (Fl_i_) at a constant rate (k_0_) into an expandable (e.g., added fluid) central compartment (V_c_) and sequentially transferred (k_12_) from Vc to a rapidly equilibrating fast peripheral compartment: V_t1_, V_F_; added fluid and then (k_23_) to a slowly equilibrating peripheral compartment (V_t2_, V_S_; added fluid). k_b_ represents non-urine fluid losses and fluid that is not eliminated from V_t_ or V_t2_ (V_S_) in the two and three compartment models. C right: The relative percent volume–time changes (ΔV) in V_c_ (solid red), V_t1_ (dashed green), and V_t2_ (solid blue) for the three compartment model.

**Figure 13:**
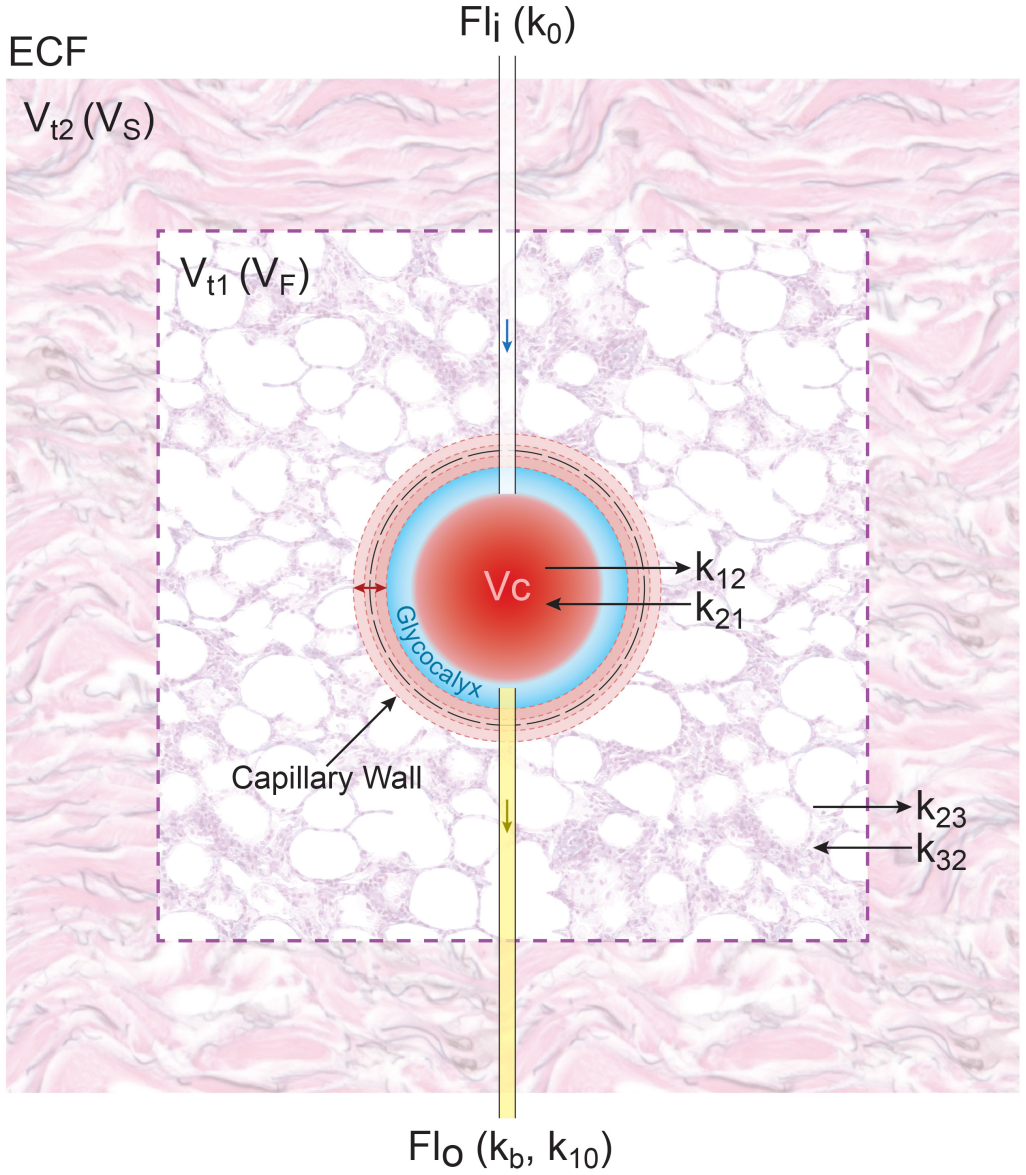
Capillary and interstitial tissue. Blood (plasma and red blood cells) contained within perfused capillaries (e.g., Vc) is separated from interstitial tissue by an endothelial surface layer that includes the glycocalyx and capillary wall (i.e., endothelial cells). Volume kinetics divides the interstitial volume into two theoretical compartments, fast (V_t1_, V_F_) and slow (V_t2_, V_S_). Micro rate constants (k) describe the rate of fluid movement out of (k12, k23) and into (k21, k32) Vc and Vt1 (VF), respectively. Once interstitial hydration rises 20%−50% above baseline values, interstitial pressure becomes positive, causing mechanical strain, as well as the disentanglement and degradation of glycosaminoglycans. This reduces matrix fiber density, increases free interstitial fluid, and enhances interstitial compliance (C_com_), “opening” a fluid reservoir or “third space” (V_t2_, V_S_). All components [e.g., capillary wall (red double-headed arrow), V_F_, and V_S_] can expand and contract (dashed lines).

**Figure 14:**
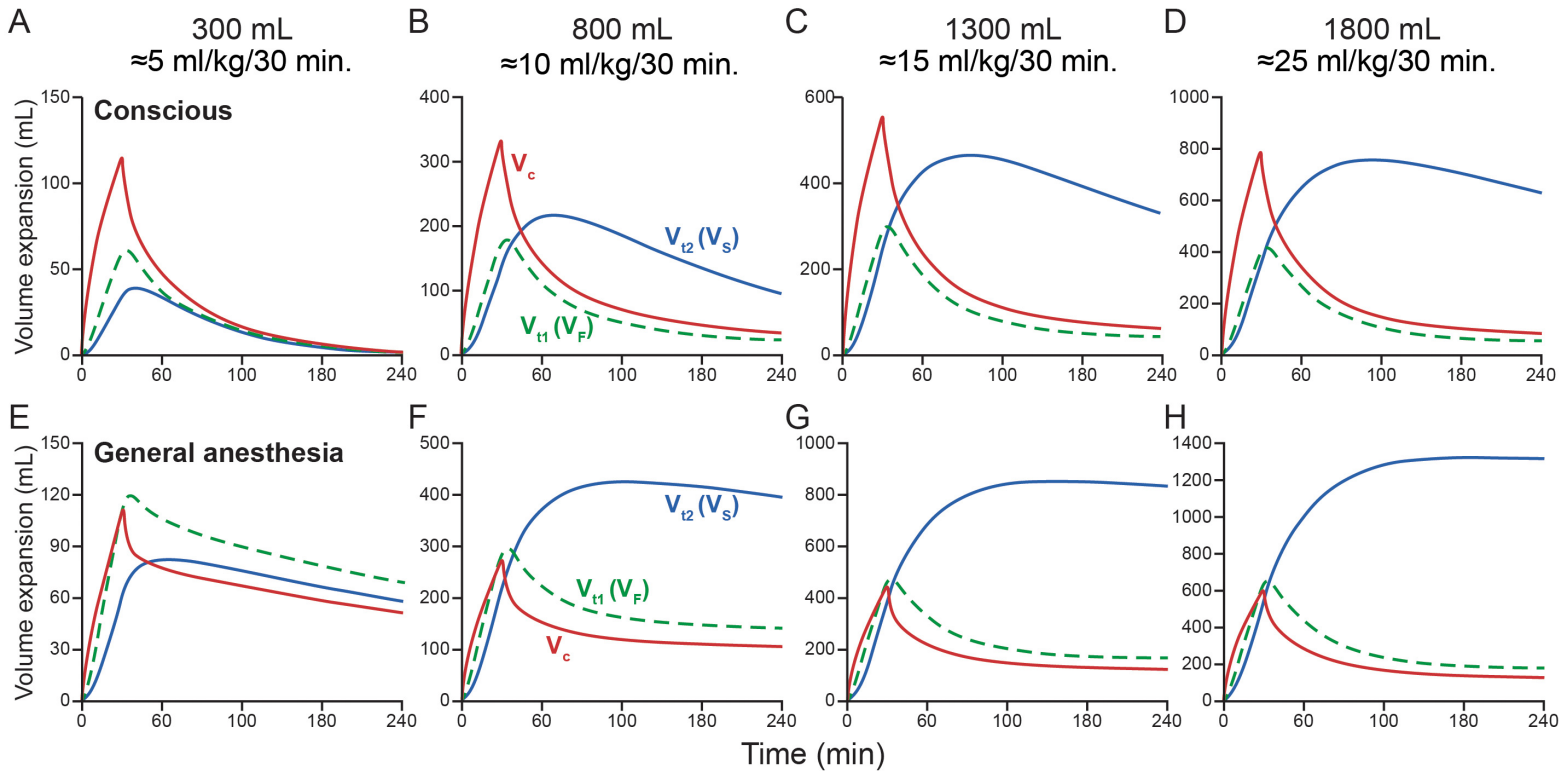
Simulations of volume–time expansion in the central (V_c_, red), fast (V_t1_, dashed green), and slow (V_t2_, V_S_, blue) fluid compartments when increasing volumes of Ringer's solution are administered over 30 min to conscious **(A–D)** and anesthetized **(E–H)** humans. Note the increase in V_t2_ (V_S_) volume and delayed elimination with increasing rates of fluid administration (modified from reference (263)).

Volume kinetic spaces represent conceptual, mathematical compartments that quantify the distribution of infused fluids based on plasma dilution and urine output. Hemoglobin and Alb dilution time data are used to determine which model (e.g., one, two, or three compartments) best fits the data (Figure 12). Most IV fluid studies suggest that IV fluid disposition can be described by one (e.g., colloids), two, or three (e.g., crystalloids) compartmental models and first-order kinetics (249, 251–253, 258–260). Variability in VK parameters among different subjects is frequently observed due to individual characteristics (e.g., weight, sex, and age), physiology (e.g., hydration, arterial blood pressure, and urine output), the effects of drugs (anesthetic, vasoactive), and health status (e.g., hemorrhage, trauma, or sepsis). The impact of these individual characteristics (i.e., covariates) on VK variables is identified by computational programs (e.g., non-linear mixed-effects modeling: NONMEM) that analyze a group of individuals (e.g., population analysis) rather than the dilution-time data of a single individual (261–264). Volume kinetic analysis has identified context- and species-specific differences in the distribution and elimination of IV fluids from Vc to fast (e.g., k_12_, k_21_; V_t1_, and V_F_) and then slow (k_23_, k_32_; V_t2_, VS) fluid compartments (Figure 13) (179, 253, 259, 260, 265–267). Compartmental modeling and covariate analysis help to characterize the disposition of different infusion rates (i.e., k_0_), fluid volumes (Figures 14A–D), general anesthesia, and awakening from anesthesia change IV fluid distribution (e.g., k_12_, k_21_, k_23_, and k_32_), elimination (e.g., k_10_), and accumulation (e.g., increased k_b_; Figures 14E–H) (259, 268–270). Volume kinetic analysis has also helped to identify the effects of adrenergic drugs on crystalloid disposition in anesthetized (268) and septic animals (269, 270). Alpha-1 adrenergic agonists accelerate fluid distribution (k_12_) and elimination (k_10_), while beta-1 stimulation prolongs these effects (269).

Approximately 75%−80% of an IV crystalloid fluid bolus moves into the interstitial space in a relatively short period (e.g., t_1/2_ 8 min.) compared to 20%−30% for conventional colloids (e.g., distribution t_1/2_ 2–6 h) (13, 257, 271, 272). This difference in intravascular retention and duration has consistently resulted in superior macro–microcirculatory hemodynamic effects for colloids compared to crystalloids (273, 274) and has resulted in a range of equi-efficacious crystalloid–colloid dosing ratios (e.g., crystalloid to colloid: 1.5–4) (275–278). Large systematic reviews, however, have provided weak or unsupportive evidence that colloids improve outcomes or reduce morbidity and mortality compared to crystalloids (279–281). Furthermore, colloid administration has been associated with coagulopathy, acute kidney injury (AKI), anaphylactoid reactions, and fluid overload in humans, raising concerns in animals due to similar biological and physiological mechanisms (282, 283).

Edema is a major prognostic indicator of morbidity and mortality in septic patients (284, 285). Fluid accumulation and overload occur more rapidly when capillary permeability and C_com_ are increased, lymphatic pumping is impaired, and urine output is decreased (e.g., due to anesthesia). Foundational physiological studies have provided guidance regarding the maximal rate and volume at which IV crystalloids can be administered before interstitial fluid accumulation becomes problematic (19, 140, 145, 156, 157, 160, 161, 163, 243, 286). Fluid accumulates in the body when the rate of fluid administration exceeds the maximal rate of sensible (e.g., urine and fecal) and insensible (e.g., evaporation from skin and breathing) losses. This rate is primarily determined by urine output in monogastrics since fecal (≈0.5 ml/kg/h) and insensible losses (e.g., ≈0.5 ml/kg/h) are comparatively low compared to ruminants and hind–gut fermenters. Urinary losses can reach values ranging from 5 to 8 ml/kg/h in dogs that are being fluid loaded (287). Fluid accumulates in the interstitium when Jv > J_L_ (2 ml/kg/h). Interstitial fluid accumulation expands the ECM (i.e., GAG expansion), reduces matrix density, and facilitates solute transport (160, 163). Once interstitial fluid volume exceeds 20%−50% (≈30–75 ml/kg) of its normal volume, the ECM begins to break down, fluid mobility increases, and a slow-turnover fluid reservoir or “third space” (V_t2_, Vs) opens, which markedly prolongs the fluid half-life (137, 145, 160, 165). Increased rates of fluid administration and disease (e.g., renal impairment, heart failure, and sepsis) would shorten the time to fluid accumulation and overload (288–290). Volume kinetic analysis provides clinically relevant insights regarding species- and context-dependent differences in fluid distribution, elimination, and central compartment volume expansion in response to different volumes and rates of fluid administration. This should facilitate more tailored fluid therapy prescriptions that optimize plasma volume expansion. For example, species or contexts that demonstrate faster distribution (k_12_) and slower redistribution (k_21_) or elimination (k_10_) suggest that more frequent administration of smaller fluid boluses is likely to be more effective than large (e.g., > 15–20 ml/kg/10–15 min) fluid boluses (13). Volume kinetic analysis does not diagnose fluid deficits, predict fluid responsiveness, tissue perfusion, tissue oxygenation, or tissue viability. Sensitive and specific monitoring modalities are required to determine if the translation of volume kinetic-derived insights enhances outcomes.

## Conclusion and future considerations

Macro–micro hemodynamic–interstitial– lymphatic concordance requires an understanding of the structure, function, and pathophysiologic processes responsible for alterations in BV, fluid circulation, fluid balance, and cardiovascular, interstitial, and lymphatic function. Optimal IV fluid therapy requires awareness of the physiologic processes that determine and regulate fluid circulation and the species-dependent context associated with alterations in fluid disposition (e.g., VK). Fluid infusion increases BV and is assumed to improve P, Q, J_s_, J_v_, and J_ox_ but can produce unfavorable responses in system concordance depending on the rate and volume of fluid administered and the timing of fluid administration. Novel strategies and monitoring methods (e.g., POCUS-directed volume status assessment) are needed to identify and address species- and context-dependent fluid therapy strategies (e.g., liberal vs. restrictive) and the timing of IV fluid prescription (291–295). A multi-faceted approach that includes fluids and therapies that maintain tissue perfusion, restore the GCX and endothelial function (289, 296–298), improve lymphatic pumping (299), and ensure controlled cellular oxygenation (300–304) is needed to improve outcomes in critical patients.

## Figures and Tables

**Table:** 

Alb	albumin
AVD	apoptotic volume decrease
BV	blood volume
CaO_2_	arterial blood oxygen content
C_cap_	capacitance
C_com_	compliance
DO_2_	oxygen delivery
DO_2crit_	critical oxygen delivery
ECFV	extracellular fluid volume
ECM	extracellular matrix
ECV	effective circulatory volume
G	conductance
Ga	arteriole conductance
Gv	venule conductance
GAGs	glycosaminoglycans
GCX	glycocalyx
Hb	hemoglobin
Hb_0_	Hb concentration at time 0
Hb_t_	Hb concentration at any time t
Hct: φ	hematocrit
IV	intravenous
J_L_	lymph transport
J_ox_	oxygen transport
Js	solute transport
Jv	fluid transport
K	filtration coefficient
l	length
Lp	hydraulic conductance
MAP	mean arterial pressure
MMPs	metalloproteinases
MVFI	microvascular flow imaging
P	hydrostatic pressure
P_c_	capillary hydrostatic pressure
P_D_(t)	plasma dilution at any time t
P_i_	interstitial hydrostatic pressure
P_lp_	lymphatic pumping pressure
Pmcf	mean circulatory filling pressure
Pmsf	mean systemic filling pressure
Pra	right atrial pressure
PtO_2_	tissue oxygen tension
PBPK	physiologically based pharmacokinetics
PK	pharmacokinetics
POCUS	point of care ultrasound
PPV	pulse pressure variation
PV	plasma volume
PV(0)	plasma volume at any time 0
PV(t)	plasma volume at any time t
PVD	perfused vascular density
PVI	pulse variability index
Q	blood flow or cardiac output
Q_c_	capillary blood flow
R	resistance
Ra	arteriole resistance
R_L_	lymphatic resistance
Rv	venous resistance
R_VR_	resistance to venous return
RVD	regulatory volume decrease
RVI	regulatory volume increase
S	surface area
SaO_2_	arterial Hb oxygen saturation
SpO_2_	peripheral oxygen saturation of Hb
SVV	stroke volume variation
TBW	total body water
TVD	total vascular density
V_Acc_	interstitial accessible volume
Vc	central compartment
V_Ex_	interstitial excluded volume
Vi	total interstitial fluid volume
VR	venous return
X_0_	initial amount of substance at time 0
X_t_	amount of substance at a specific time t
e	Euler's number (approx. = 2.718)
Δ	change in quantity
η	viscosity
σ	membrane reflection coefficient
π	Pi (e.g. constant = 3.141)
π_c_	capillary oncotic pressure
π_g_	sub-GCX oncotic pressure
π_i_	interstitial oncotic pressure
ϕ	hematocrit
Ω	geometric constant.

## Footnotes

1
https://www.fda.gov/regulatory-information/search-fda-guidance-documents/classification-products-drugs-and-devices-and-additional-product-classification-issues

2
https://www.fda.gov/vaccines-blood-biologics/blood-bloodproducts#::text=Related%20Information%20%20Blood%20Research.%20%20Biologics%20Products%20&%20

3
https://www.fda.gov/vaccines-blood-biologics/safety-availability-biologics/labeling-changes-mortality-kidney-injury-and-excess-bleeding-hydroxyethyl-starch-products

4
https://www.fffenterprises.com/assets/downloads/Article-FDASafetyCommunicationBoxedWarning6-13.pdf

5
https://www.fda.gov/drugs/drug-approvals-and-databases/drugsfda-golassary-terms

6
https://www.fda.gov/regulatory-information/search-fda-guidance-documents/bioequivalence-studies-pharmacokinetic-endpoints-drugs-submitted-under-abbreviated-new-drug
